# Improved taxonomic assignment of human intestinal 16S rRNA sequences by a dedicated reference database

**DOI:** 10.1186/s12864-015-2265-y

**Published:** 2015-12-12

**Authors:** Jarmo Ritari, Jarkko Salojärvi, Leo Lahti, Willem M. de Vos

**Affiliations:** Department of Veterinary Biosciences, University of Helsinki, Helsinki, Finland; Laboratory of Microbiology, Wageningen University, Wageningen, the Netherlands; Department of Bacteriology and Immunology, University of Helsinki, Helsinki, Finland

**Keywords:** Next-generation sequencing, 16S, Ribosomal RNA, Human intestinal microbiota, Bacteria, Archaea, Taxonomy

## Abstract

**Background:**

Current sequencing technology enables taxonomic profiling of microbial ecosystems at high resolution and depth by using the 16S rRNA gene as a phylogenetic marker. Taxonomic assignation of newly acquired data is based on sequence comparisons with comprehensive reference databases to find consensus taxonomy for representative sequences. Nevertheless, even with well-characterised ecosystems like the human intestinal microbiota it is challenging to assign genus and species level taxonomy to 16S rRNA amplicon reads. A part of the explanation may lie in the sheer size of the search space where competition from a multitude of highly similar sequences may not allow reliable assignation at low taxonomic levels. However, when studying a particular environment such as the human intestine, it can be argued that a reference database comprising only sequences that are native to the environment would be sufficient, effectively reducing the search space.

**Results:**

We constructed a 16S rRNA gene database based on high-quality sequences specific for human intestinal microbiota, resulting in curated data set consisting of 2473 unique prokaryotic species-like groups and their taxonomic lineages, and compared its performance against the Greengenes and Silva databases. The results showed that regardless of used assignment algorithm, our database improved taxonomic assignation of 16S rRNA sequencing data by enabling significantly higher species and genus level assignation rate while preserving taxonomic diversity and demanding less computational resources.

**Conclusion:**

The curated human intestinal 16S rRNA gene taxonomic database of about 2500 species-like groups described here provides a practical solution for significantly improved taxonomic assignment for phylogenetic studies of the human intestinal microbiota.

**Electronic supplementary material:**

The online version of this article (doi:10.1186/s12864-015-2265-y) contains supplementary material, which is available to authorized users.

## Background

As the most genetically diverse and functionally complex microbial ecosystem of the human body the intestinal microbiota has become one of the major areas of interest in microbial ecology [1]. In particular, efforts have been undertaken to understand how individual composition and variation of the microbiota together with host genetic and environmental factors influence human health [2, 3]. Over the past decade it has become evident that the microbiota exerts various beneficial effects to the host physiology during the development and in adulthood, notably through immunity and nutrition [4, 5], and deviations from a balanced microbial composition are related to systemic problems, such as diabetes, obesity and allergy [6–8]. Progress in molecular analysis of the microbiota has been made possible largely by the advance of next-generation sequencing technology, which has allowed studying the composition and dynamics of microbial communities with unforeseen scale and resolution [9, 10].

The bacterial and archaeal 16S small subunit ribosomal RNA (16S rRNA) gene has been established as the most widely used phylogenetic marker due to its conserved and variable regions and universal presence in prokaryotes. By sequencing the pool of 16S rRNA genes, community composition can be investigated in a comprehensive and rapid manner by high-throughput sequencing platforms harbouring the capacity for millions of reads per single run [11, 12]. As a result of increasing read length, sample multiplexing capability and reducing costs, 16S sequence data is being accumulated from various microbial ecosystems, and vast reference databases like Silva [13, 14], Greengenes (GG) [15] and RDP [16] have been built to enable phylogenetic analysis of high-throughput data.

While being highly successful at gathering data, the high-throughput technologies also present challenges for data analysis by requiring sophisticated computational methods not only in correcting technical artifacts but also for organizing the output and extraction of biologically meaningful features. A crucial step in deciphering 16S rRNA reads data is the taxonomic annotation of the discovered sequences. This holds true especially because current sequencing technologies typically cover only a part of the 16S rRNA gene, the large number of reference sequences and limited resolution at genus and species levels [17, 18]. Taxonomic annotations have been shown to depend on several factors, including sequence length, target region of the 16S gene, OTU classification method and assignment algorithm. Although many comparative studies have addressed these technical factors [19–22], the effect of the reference database on the accuracy of taxonomic assignment remains less well known. The standard approach has been to use as comprehensive a database as possible to minimize the number of unclassified sequences [23]. However, increasing database size also makes it potentially more difficult to assign taxonomy at genus and species levels as the likelihood of ambiguous assignment increases due to larger number of competing sequences in the search space. On the other hand, better taxonomic resolution would be valuable in profiling the human gut microbiota because different species and genera can associate with different conditions and outcomes [24]. Furthermore, the 16S rRNA gene has been shown to have considerably higher ambiguous assignment rate at lower taxonomic levels compared with other taxonomic marker genes [18], making its use somewhat problematic despite extensive reference data sets.

We hypothesized that by reducing the size of the reference database to encompass only the sequences innate to the environment under study would lead to improved taxonomic assignations at lower taxonomic levels due to less competition among targets. In this respect, the human intestinal microbiota presents an advantageous model system because it is already well characterized by sequencing [25, 26] while genus and species level taxonomic assignment of new sequencing data remains challenging [17]. Moreover, a curated set of over 1000 cultured bacterial and archaeal species from the human intestinal ecosystem has recently been reported [27]. To this end, we constructed a custom human intestinal 16S rRNA database, termed HITdb, including all currently known cultivable gastrointestinal prokaryotes as well as operational taxonomic units (OTUs) generated from high-quality 16S rRNA sequences originating from human intestinal tract. Here, we have evaluated the taxonomic assignment performance of the custom database by comparing it with the current standard, the Greengenes database, and demonstrate that the custom database improves taxonomic assignation of human intestinal 16S high-throughput reads. The 2473 species-like 16S rRNA sequences present in the HITdb also provide a minimum estimate for the number of species present in the human intestinal ecosystem.

## Results and discussion

**Fig. 1:**
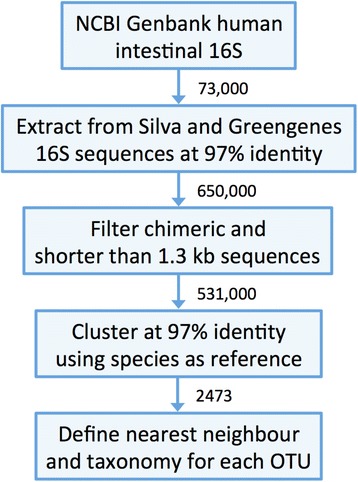
Main steps in the construction of the human intestinal tract 16S taxonomic database (HITdb). Human intestinal specific sequences were pulled down from the Greengenes and Silva databases using Genbank sequences. Obtained sequence data were clustered at 97 % identity by using cultivable human intestinal species as a reference. A cultivable nearest neighbour was determined for each OTU. The taxonomic lineages were determined based cultivable species taxonomy, Greengenes and manual curating

To construct the human intestinal microbiota 16S rRNA database (HITdb), we extracted a subset from Greengenes and Silva databases by using a set of over 73,000 NCBI GenBank 16S rRNA sequences annotated as originating from the human gastrointestinal tract. Pulling down the human intestinal subset from Silva and Greengenes by matching the GenBank sequences at 97 % global similarity (used as an OTU definition throughout this work) resulted in over 650,000 sequences, which were further filtered from potential chimeras and shorter than 1.3 kb sequences to the final number of 531,712 sequences (Fig. 1). Clustering the sequences using presently known cultivable species [27] as reference resulted in altogether 1482 bacterial and 27 archaeal *de novo* OTUs. In total the database contained 2473 species-like clusters (Fig. 1). By including only curated and near full-length sequences and requiring at least two sequences per cluster (i.e. nonsingletons) we aimed to minimize the possibility of generating spurious OTUs, which are prone to occur with short or chimeric sequences [28, 29]. Each *de novo* OTU represents at least 3 % sequence identity difference to other OTUs and known species. Although the 3 % is only an arbitrary threshold and differences in genetic distances between taxonomic groups vary so that OTUs may not be monophyletic [30, 31], it is commonly accepted as an approximate species assignment in 16S analysis [32, 33]. Defining the OTUs by sequence identity is to some extent facilitated by using near full-length 16S sequences, which provide more robustness in contrast to smaller fragments of the rRNA gene where the application of the 97 % rule would become more problematic. Although other clustering methods exist that show improvement relative to a strict identity cutoff based OTU definition [30, 34], they tend to be expensive in terms of required computational resources and thus challenging for processing large (i.e. > 10^5^ sequences) datasets like in this study. For example, the heuristic OTU clustering algorithm Uclust applied here to construct HITdb is slightly less robust than the UPGMA method [31] but efficient with large datasets.

Finally, the HITdb sequences were taxonomically assigned based on the cultivated species’ taxonomy, Greengenes and manual curating. A nearest neighbour cultivable species was determined for each OTU to facilitate the interpretation of the OTUs. Phylogenetic trees constructed from bacterial and archaeal sequences (Additional file 1) were found to correspond with the nearest neighbour information.

In order to evaluate how comprehensively the HITdb represents taxonomic diversity we performed a computational rarefaction analysis based on the sequence data used for constructing the HITdb (see Methods). The obtained rarefaction curve shows that the number of 97 % OTUs is not quite saturated at current sequence data (Additional file 2), which would indicate that the full species-level diversity is not fully covered. On the other hand, actual rarefaction by sampling random subsets from the sequence data, defining OTUs for each sampled subset and calculating the number of known unique species and genera represented by the OTU clusters showed that the numbers were not significantly lower in samples constituting about 80 % of the original sequence data (Additional file 3), suggesting that the data is close to reaching saturation. Altogether, these results suggest that the HITdb is able to capture the diversity of known taxa quite well, because in all tests with actual rarefaction the observed numbers would be expected to drop significantly if the sampling depth was a limiting factor. However, the sequence coverage in clusters representing known species was typically higher than in OTUs (data not shown), which could explain why saturation is seen with known species in actual rarefaction, but not when all OTUs are considered in computational rarefaction. Since most of the 16S sequences are assigned to clusters of known species, it also implies that if some species-like groups were missing from HITdb, they would be increasingly rare and therefore probably not highly relevant.

The number of entries in the curated HITdb, *viz.* a total of 2473, can be seen as the present estimate for the minimal number of species expected to be present in the human intestinal tract. Since according to the rarefaction analysis the number of OTUs is probably not quite saturated yet, the number may still increase with new data. However, there may be only a limited number of new OTUs emerging, similar to the situation with metagenomic data that shows only a limited increase to the known information pool with the addition of new metagenome sequences [35]. In any case, an earlier estimate of about 1800 human intestinal species remaining uncultured [36] is consistent with HITdb because the number of cultured species has increased since then [27], leaving about 1500 species still uncultured. This is an important estimate to keep in mind when designing strategies to culture the not-yet cultured species from the human intestinal tract.

**Fig. 2:**
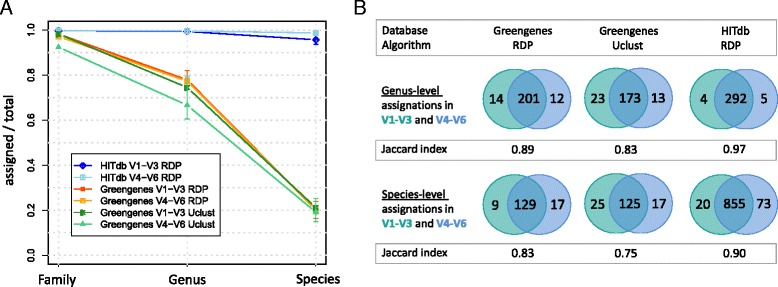
Taxonomic assignment of synthetic reads. **a** Comparison of numbers of family, genus and species level assignments relative to missing assignments at same levels between HITdb and Greengenes databases. Taxonomy from Greengenes has been assigned with both RDP and Uclust algorithms, while taxonomy from HITdb has been assigned solely with RDP. The error bars represent upper and lower limits of 1000 bootstraps. **b** Venn diagrams showing genus and species level assignations between V1-V3 and V4-V6 synthetic reads for Greengenes and HITdb. The database and algorithm are indicated in columns and the taxonomic level in rows. The green and blue circles represent 16S rRNA gene regions V1-V3 and V4-V6, respectively. Jaccard index shows intersection relative to union for each diagram. The numbers represent absolute counts of assigned taxa

To assess the performance of HITdb in taxonomic assignment we compared it with Greengenes by analysing synthetic data constructed from the sequences of known human gut resident species (Additional file 4). Since the taxonomic position of the synthetic reads is known, it was possible to evaluate the effect of the used database without potential confounding factors such as sequencing technology or unknown sequence content that might favour one database over another. We first determined the relationship between assigned and unassigned taxa at different taxonomic levels for the two tested databases (Fig. 2a). The results show that the relative numbers of missing genus-level assignations was below 80 % with Greengenes, irrespective of used 16S region or assignment algorithm, while with HITdb there were practically no missing assignments observed. At the level of species the difference was even more pronounced since with Greengenes only about 20 % of synthetic reads could be assigned to species-level, while with HITdb over 90 % assignation rate was achieved. Similar results were obtained by using Mothur as the assigner algorithm and Silva as the reference database, although with Silva the assignment rate at all tested levels was extremely low (Additional file 5) and thus Silva was not included in further comparative analyses. It should be notified that although HITdb contains the synthetic read parent sequences, the same should also be true for Greengenes because only known species were included in the analysis, so potentially missing taxa cannot explain the observed difference. The assigner algorithm is also not likely to be explanatory for this result because three different approaches were compared; naïve Bayesian (RDP) [37] and Mothur [34], and sequence similarity majority vote based Uclust (as part of the Qiime pipeline).

**Fig. 3:**
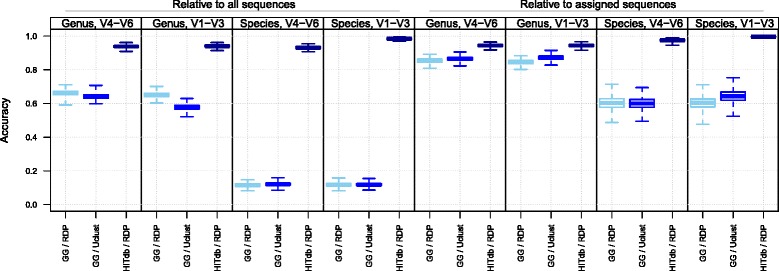
Taxonomic assignment accuracy. Proportions of correctly assigned synthetic reads relative to the total number of assignments (left) and relative to assigned sequences only (right). The 16S sequence regions V1-V3 and V4-V6 are shown separately at genus and species levels in Greengenes and HITdb. The box plots represent variation over 1000 bootstraps

Secondly, we compared the agreement of taxonomic assignations between two different regions of the 16S rRNA gene, V1-V3 and V4-V6, by using GG and HITdb. At both genus and species levels the number of shared assignments relative to all assignments was better with HITdb, as shown by the Jaccard index and absolute numbers of shared assignments (Fig. 2b). Moreover, accuracy estimation of synthetic read assignments between Greengenes and HITdb showed that HITdb performs better in terms of absolute and relative correct assignments (Fig. 3). These results further suggest that the database itself is a major determinant in genus and species level assignment performance. This is an important observation, as one of the limitations in current intestinal microbiota analysis is the assignment at the species level and its subsequent interpretation.

**Fig. 4:**
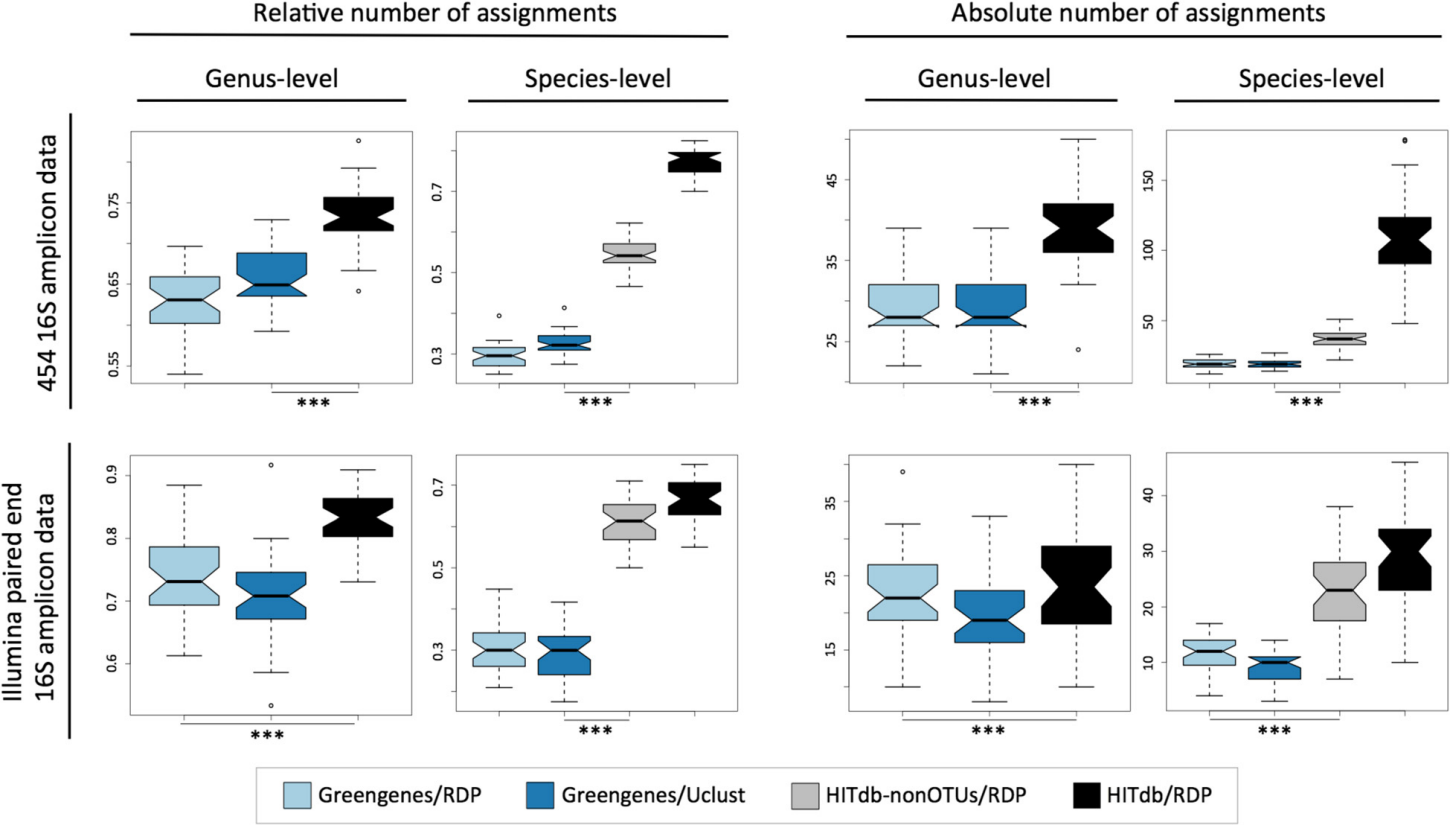
Comparison between Greengenes and HITdb using data from biological samples. The used data set is indicated in rows, and relative and absolute numbers of assignments at genus and species levels in columns. At species level, the results for HITdb additionally show the biological species only (i.e. without OTUs) for easier comparison with Greengenes. ***p < 0.001. *n* = 119 and *n* = 40 samples for 454 and Illumina data sets, respectively

To further evaluate the performance of HITdb we analysed high-throughput 16S amplicon reads data generated from two sets of fecal samples by pyrosequencing and paired-end Illumina technologies (Fig. 4). We found that the number of species and genus level assignations was higher in both absolute and relative terms with HITdb in contrast to Greengenes. Since Greengenes does not contain identifiable OTUs (but just missing taxonomic information for unknown groups), we removed the OTUs from HITdb assignation results and included only known species (indicated by gray boxplots in Fig. 4). Also in these comparisons the number of assigned taxa was significantly higher with HITdb, further confirming the observation that the database search space size itself is likely to be an important factor in taxonomic assignment. To rule out other biases caused by different numbers of non-assigned reads, we compared the numbers of reads not assigned to any phylum in HITdb and Greengenes. The numbers of non-assigned reads in HITdb assignations were at least as low as in Greengenes, amounting to approximately 0.1 % of total (Additional file 6). This indicates that the HITdb enables comprehensive assignment in our set of test samples in a 16S region and sequencing technology independent manner, and in general suggests that HITdb does not limit taxonomic assignment despite being much smaller in content than Greengenes.

**Fig. 5:**
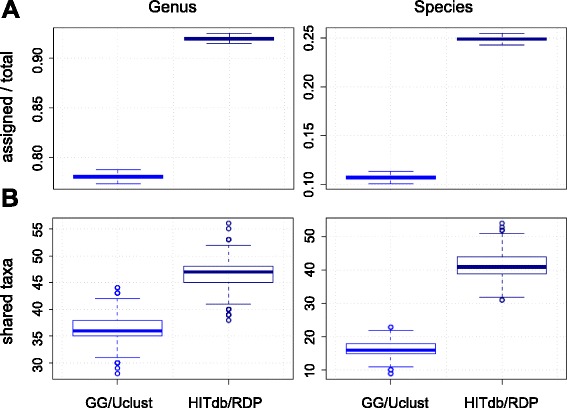
Analysis of Human Microbiome Project data. **a** Numbers of genera and species found by Greengenes and HITdb in HMP 16S samples (*n* = 192). **b** Numbers of shared genera and species between HMP shotgun metagenomics profiling and 16S analysis of the same HMP samples by Greengenes and HITdb (*n* = 23). The boxplots represent variation over 1000 bootstraps

To confirm the results on biological data with an independent data set and methodology, we performed comparative tests using publicly available Human Microbiome Project (HMP) data. HITdb performance was first compared with Greengenes in 192 fecal 16S sequencing samples (Additional file 7). The results indicate that HITdb was able to detect a higher number of genera and species than Greengenes from these data as well (Fig. 5a), being consistent with results from other data sets analysed in similar way. Furthermore, we compared taxonomic profiles from our 16S analysis with shotgun metagenome taxonomic profiling available in HMP. The numbers of genera and species shared with the metagenomic profiles were again higher in HITdb than in Greengenes (Fig. 5b), suggesting better accuracy for HITdb. All in all, the comparative test results suggest that HITdb outperforms Greengenes in quantity and quality independently of data source.

**Fig. 6:**
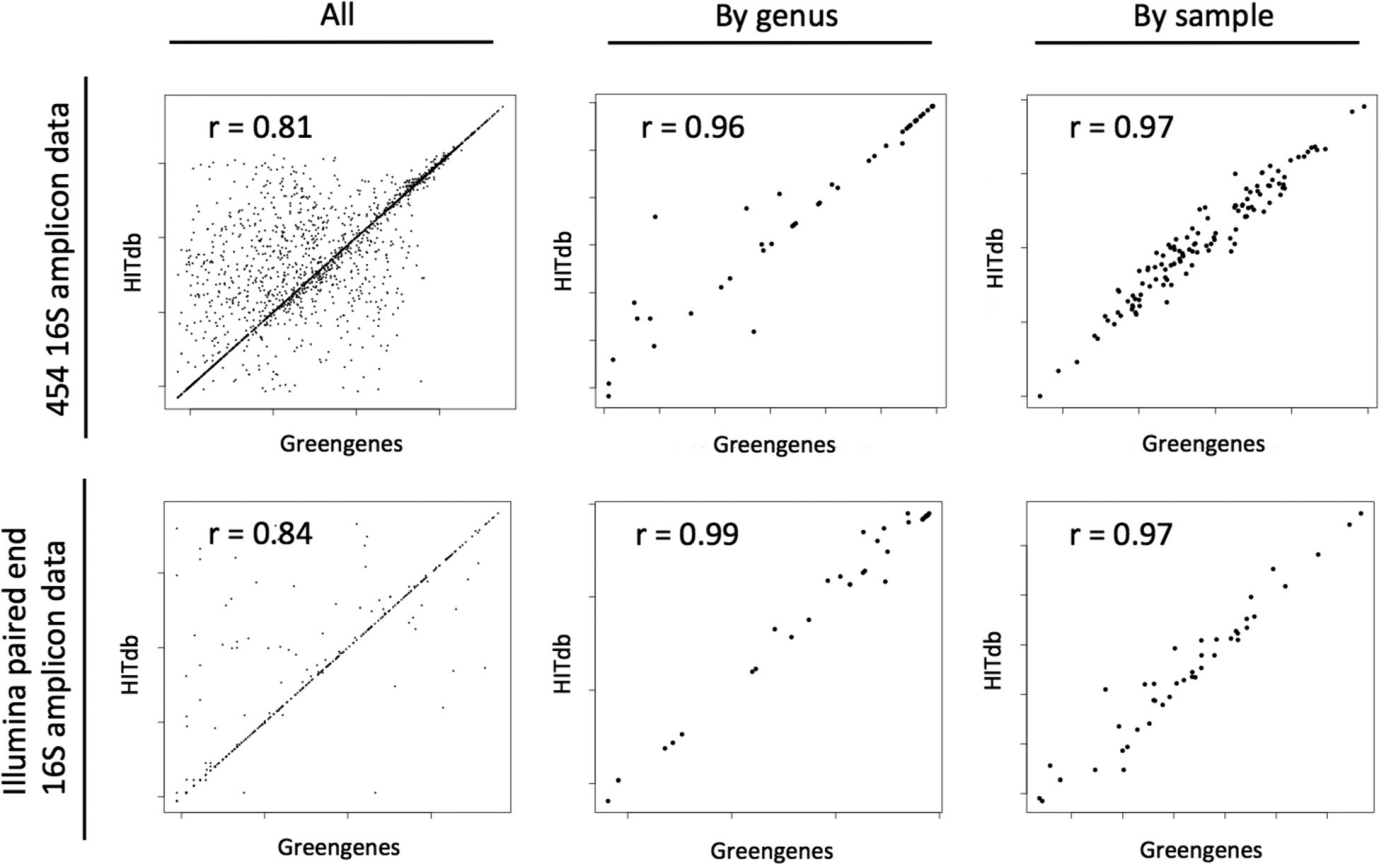
Correlation of relative abundances between Greengenes and HITdb. The data points represent log relative abundances of common genera between Greengenes and HITdb from 454 and Illumina 16S amplicon data sets. The data are summarised over samples for each genus (“by genus”) and summarised over genera for each sample (“by sample”). The correlation coefficients are Pearson’s

We also tested how well the relative numbers of taxa correlated between HITdb and Greengenes (Fig. 6). When considering genera averaged over all samples, or samples averaged over all genera, the Pearson correlation coefficients were over 0.95. Moreover, when considering all data points (i.e. single genus in single sample), the correlation coefficient was over 0.8, showing that HITdb largely agrees with Greengenes in a quantitative manner. Differences are also quite symmetrical over the diagonal meaning that HITdb doesn’t have tendency to systematically over- or underrepresent taxa abundances relative to Greengenes.

Computational resources required by taxonomic annotation depend on the reference database, assignment algorithm and the number of sequences to be assigned. We found that HITdb with RDP classifier is both faster and takes less memory than Greengenes using Uclust or RDP classifiers (Additional file 8). Although Uclust is very fast, it is quite memory intensive, while RDP (and Mothur) consume less memory but are slower. Since HITdb performs faster and with less memory than either algorithm with Greengenes, it may be expected to scale well for increasingly large datasets for future needs.

## Conclusions

Profiling the composition of intestinal microbiota relies on accurate taxonomic annotation of sequencing reads. However, large reference databases may not be able to provide optimal species- and genus-level resolution due to increasing competition in the search space. To improve low-level resolution without abandoning comprehensiveness in taxonomic assignation we propose using a dedicated reference database specific to the ecosystem under study. By constructing a human intestinal 16S database and comparing its performance with Greengenes we found that the dedicated reference database improves the assignment rate at genus and species level, suggesting that large search space may be a limiting factor in low-level taxonomic assignment. Our study provides a practical solution with considerable performance improvements, is readily applicable in human gut microbiota profiling studies and paves the way for developing similar focused databases for other model systems.

## Methods

### HITdb construction

To create a reference data set the 16S sequences of cultivable bacterial and archaeal human intestinal resident species [27] were obtained from NCBI Genbank. In addition, a Cyanobacteria related *Melainabacterium* species from curated metagenome [38], a cultivable representative of phylum TM7 from human oral cavity [39], *Intestinimonas butyriproducens* and *Methanomassiliicoccus intestinalis* [40] were included to the reference set of known species.

To obtain a comprehensive set of near full-length 16S sequences originating from human intestinal microbiota a search was performed against the NCBI Genbank nucleotide database using the command *((“Homo sapiens”[Organism] OR human[All Fields]) AND (intestinal[All Fields] OR gut[All Fields]) AND 16S[All Fields]) AND (“bacteria”[porgn] OR “archaea”[porgn]) AND 1000:2000[SLEN]*. The extracted sequences were matched against the Greengenes 13_5 [15] and Silva [13, 14] (SSURef_NR99_115_tax_silva_trunc) 16S databases at 97 % identity using Usearch v. 7.0.1001 command *usearch_global*. The matched sequences were extracted from both databases and subjected to chimeric sequence removal by UCHIME v. 7.0.1001 (command *uchime_ref*; default parameters) using the 16S reference database available at http://drive5.com/uchime/gold.fa [41]. The non-chimeric sequences were length filtered to exclude sequences shorter than 1.3 kb. The filtered sequence data was then clustered to OTUs using the cultivable species’ sequences as a reference but allowing non-matching sequences to cluster *de novo*. At minimum two sequences were required for each *de novo* OTU. The OTU clustering was performed at 97 % identity threshold in Qiime v. 1.8.0 [42] using the command *pick_open_reference_otus.py* with parameters suppress_taxonomy_assignment, min_otu_size = 2, prefilter_percent_id = 0.0, percent_subsample = 0.1 and suppress_align_and_tree. Next, the representative sequences of OTU clusters were matched back to the reference species’ sequences using Usearch v. 7.0.1001 command *usearch_global* with parameters id = 0.5 and maxhits = 1. OTUs having a match over 97 % similar to any of the cultivable species were removed (i.e. collapsed with the corresponding species). Furthermore, for each OTU, the nearest cultivable species was determined from the sequence match results. The final sequence content of HITdb consisted of representative sequences of the processed *de novo* OTUs and cultivable species.

HITdb OTU representative sequences were assigned taxonomy from the taxonomy of known species and Greengenes by using RDP classifier. In cases where HITdb was able to give a lower level assignation than Greengenes, and where all taxonomic levels were in agreement between HITdb and Greengenes, the OTU was assigned the taxonomy given by the known species. The lineages of species and OTUs were manually checked for consistency, and to adapt them to current naming convention as well as possible.

### Phylogeny

The HITdb bacterial and archaeal sequences were separately aligned using Muscle v. 3.8.31 with default settings [43]. The alignments were filtered in Qiime v. 1.8.0 using command *filter_alignment.py* with parameter suppress_lane_mask_filter. Newick formatted phylogenetic trees were built from the filtered alignments using FastTree [44]. The trees were visualized with FigTree v. 1.4.0 (http://tree.bio.ed.ac.uk/software/figtree*/*).

### Synthetic reads

The 16S sequences of 953 cultivable human intestinal bacterial species were aligned to 518R [45] and 338R [46] primer sequences allowing 2 or 3 mismatches, respectively, in order to extract V1-V3 and V4-V6 gene regions from the sequences. For V1-V3, target sequence from its start until the end of 518R alignment position was extracted. For V4-V6, sequence from 338R alignment start position until 500 bp downstream of the target was extracted. The synthetic read sequences are given in Additional file 4.

### Taxonomic assignments

Both biological and synthetic 16S reads were taxonomically assigned using in-built functions of Qiime v. 1.8.0 (*assign_taxonomy.py*, *make_otu_table.py, summarize_taxa_through_plots.py*) [42] with default parameters except for reference database where in addition to Greengenes 13_5 [15] also HITdb and Silva were used, and assignment algorithm where RDP [37] and Mothur [34] were used along with the default Uclust.

### Biological samples and 16S amplicon sequencing

Two sets of fecal samples obtained from children were sequenced for evaluating the HITdb performance with real data. Sample collection and DNA extraction were performed as described before [48, 49]. For data set 1 (119 samples) [47], PCR amplicons from bacterial 16S rRNA gene region V4-V6 were generated with forward (5’-AYTGGGYDTAAAGNG-3’) and reverse (5’-TGCTGCCTCCCGTAGGAGT-3’) primers. For data set 2 (40 samples) [48], amplicons from V1-V3 region were generated with forward (5’-AGAGTTTGATCMTGGCTCAG-3’) and reverse (5’-GTATTACCGCGGCTGCTG-3’) primers. The PCR primers contained 18-mer overhangs added to the 5’ ends [49]. Replicate PCR products were pooled and purified with Agencourt AMPure XP magnetic beads (Agencourt Bioscience) and subjected to a second PCR round with barcoded forward primers and a reverse primer, both of which attached to the respective 18-mer overhang sequences from the primers of the first PCR amplification. Phusion polymerase (Thermo Fisher Scientific/Finnzymes) with HF buffer and 2.5 % DMSO were used. Cycling conditions for both PCR reactions consisted of an initial denaturation at 98 °C for 30 s, followed by 15 cycles at 98 °C for 10 s, 65 °C for 30 s, and 72 °C for 10 s, and then a final extension for 5 min. Between 3.6 and 60 ng of template DNA were used in the initial reaction. DNA concentration and quality were measured with Qubit (Invitrogen) and Bioanalyzer 2100 (Agilent). Sample set 1 was sequenced on 454 FLX Titanium instrument and set 2 in paired-end mode (R1 = 326 bp, R2 = 286 bp) on Illumina MiSeq instrument with standard library preparation protocol. Sequencing was carried out at the DNA sequencing and genomics laboratory, Institute of Biotechnology, University of Helsinki, Finland.

### Pre-processing of 16S amplicon sequencing data

The raw pyrosequencing reads were subjected to reference-based chimera filtering using UCHIME v. 7.0.1001 [41] (command *uchime_ref*; default parameters) with 16S reference database available at http://drive5.com/uchime/gold.fa. The non-chimeric reads were length filtered to exclude reads shorter than 500 nt. Thereafter the read numbers were rarefied by randomly sampling the lowest common read number (4246) from each sample using the Biostrings library [50] in R v. 3.1.1 [51]. The reads from paired-end Illumina MiSeq sequencing data set were treated in a similar manner, except for merging of read pairs which was performed using Usearch v. 7.0.1001 command *fastq_mergepairs* [52] with parameters fastq_truncqual = 4, minhsp = 9, fastq_minovlen = 10, fastq_maxdiffs = 3 and fastq_minmergelen = 440. The quality filtering of the merged read pairs was done with Usearch *fastq_filter* with parameters fastq_truncqual = 10 and fastq_maxee = 0.75. The merged and filtered Illumina reads were rarefied to 13,303 reads per sample.

### HMP data analysis

16S data of 192 Human Microbiome Project fecal samples were obtained from Sequence Read Archive (http://sra.dnanexus.com/). The SRA sample ID codes are given in Additional file 7. The data were preprocessed as described above, except for using minimum sequence length cutoff of 400 nt and rarefaction cutoff of 4000 reads. The data were analysed in Qiime v. 1.9 with default parameter settings. The data were taxonomically assigned by Uclust and Greengenes v.13_8, and by HITdb and RDP classifier in Qiime.

Metagenomic, taxonomically profiled data from HMP were obtained from HMSMCP - Shotgun MetaPHlAn Community Profiling (http://www.hmpdacc.org/HMSMCP/). The samples with the same SRS codes as in 16S HMP data were selected for taxonomic comparison. For HITdb, OTUs were excluded from comparative analysis at species level to make comparisons equal.

### Statistical methods

In order to estimate completeness of sequence data used to define the HITdb OTUs, sampling from multinomial distribution was performed. The number of sequences binned to each species-like cluster constituted the event probabilities of the multinomial model. For each draw from the multinomial, an OTU was accepted to be present if at least two reads were binned to it. The number of OTUs was calculated from 5000 draws. The sample size was varied starting from the number of all sequences (531,442) to lower numbers at a decrement of 10,000, and the mean number of OTUs over 5000 draws was calculated for each sampling. Quantiles for OTU numbers in each sample of 5000 draws was calculated for probabilities 0.95 and 0.05.

To estimate the sampling distribution of numbers of assigned taxa in synthetic reads data, the results of taxonomic assignment were bootstrapped 1000 times for each taxonomic level and employed database/assignment algorithm combination at that level. The proportion of present vs. absent taxa was calculated for each bootstrap sampling iteration.

To compare absolute and relative numbers of assigned taxa between databases in 454 and Illumina sequencing data sets, paired two-way Wilcoxon signed rank test was performed. All analyses were performed in R software v. 3.1.1 [51].

### Availability of supporting data

HITdb is available in GitHub at https://github.com/microbiome/HITdb.git.

For direct download, use https://github.com/microbiome/HITdb/archive/master.zip.

The contained README file provides instructions and other information.

## Additional files

Additional file 1:
**Phylogenetic trees.** Package containing Newick and figure files of bacterial and archaeal phylogenies in HITdb. (PDF 22521 kb) Additional file 2:
**Computational rarefaction.** The figure shows the mean number of OTUs calculated from 5000 draws at different sample sizes from multinomial distribution. The dashed lines indicate the 0.95 and 0.05 quantiles. The horizontal red dotted line marks the number of all found clusters in the original data. (PDF 71 kb) Additional file 3:
**Rarefaction based on known taxa.** The figure shows the numbers of taxa calculated from samples of sequence data used for constructing the HITdb. Boxplots showing the numbers of found species (A) and genera (B) at two sample sizes (about 90 % and 80 % of sequences, *n* = 9 and *n* = 10, respectively). The horizontal red dashed line shows the number of OTUs in all sequences (100 % of sequences). (PDF 52 kb) Additional file 4:
**Synthetic reads.** A table listing the V1-V3 and V4-V6 16S sequences from known human intestinal bacterial species used in the study. (XLS 1406 kb) Additional file 5:
**Assignment of synthetic reads.** The figure shows the relative numbers of assigned taxa for synthetic reads using Silva, Greengenes and HITdb databases, and RDP, Uclust and Mothur algorithms. (PDF 5 kb) Additional file 6:
**Proportion of missing taxonomic assignments.** The figure shows taxonomic assignments missing from Phylum level downwards in Greengenes and HITdb. The data is from two sets of biological samples sequenced either with 454 or Illumina MiSeq. (PDF 91 kb) Additional file 7:
**SRA ID codes of HMP samples.** A Table listing the Sequence Read Archive accession codes of the Human Metaproteome Project samples used in the study. (XLS 30 kb) Additional file 8:
**Comparison of memory and time usage.** The figure shows memory and time usage in HITdb and Greengenes databases. The measurement was done using 937 V4-V6 sequences from human intestinal bacterial species. (PDF 4 kb)
