# Supplementary figures and images for: Improved taxonomic assignment of human intestinal 16S rRNA sequences by a dedicated reference database
Source: BMC Genomics. 2015 Dec 12;16:1056. doi: 10.1186/s12864-015-2265-y (PMC4676846; doi:10.1186/s12864-015-2265-y)

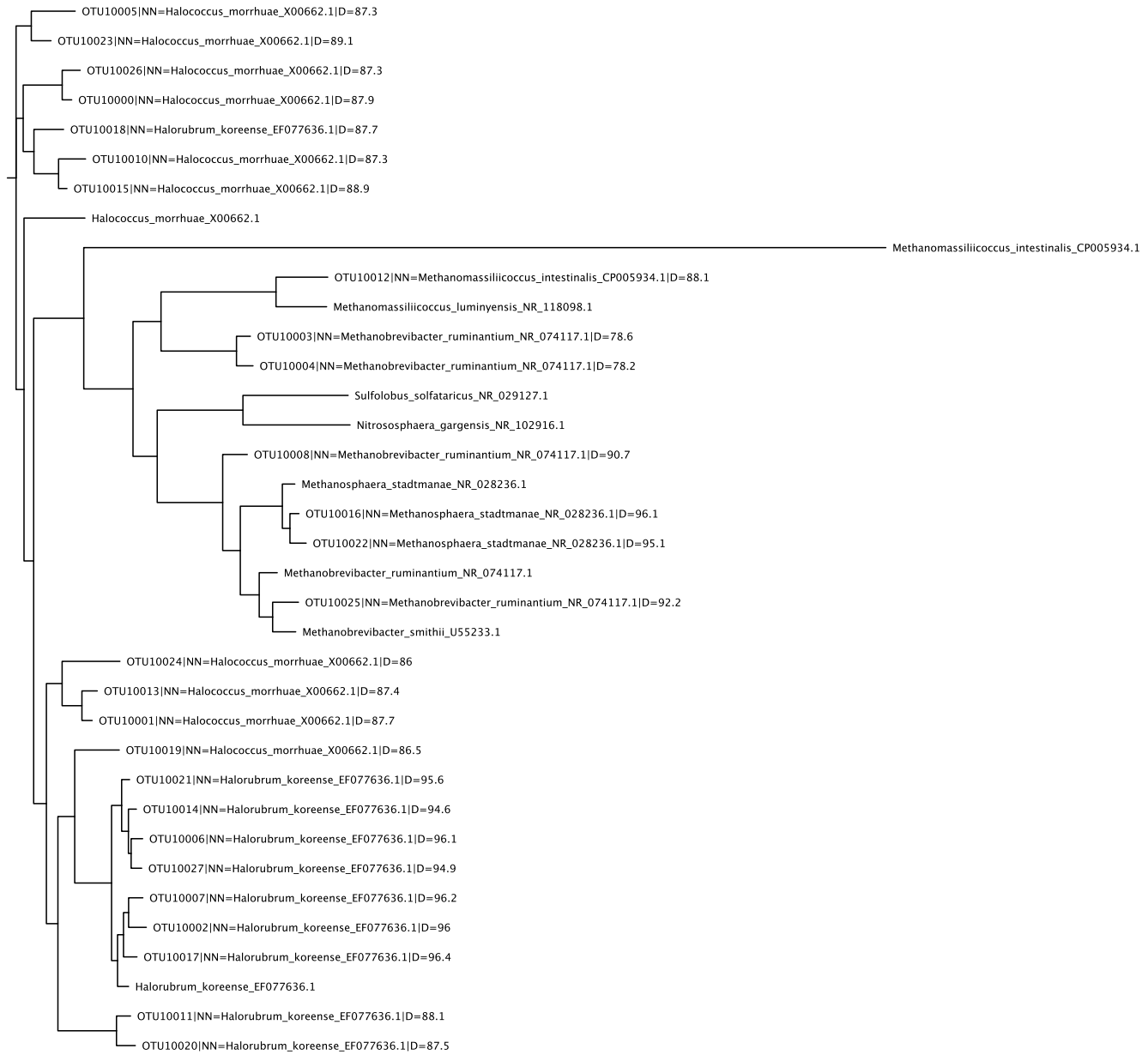

0.2

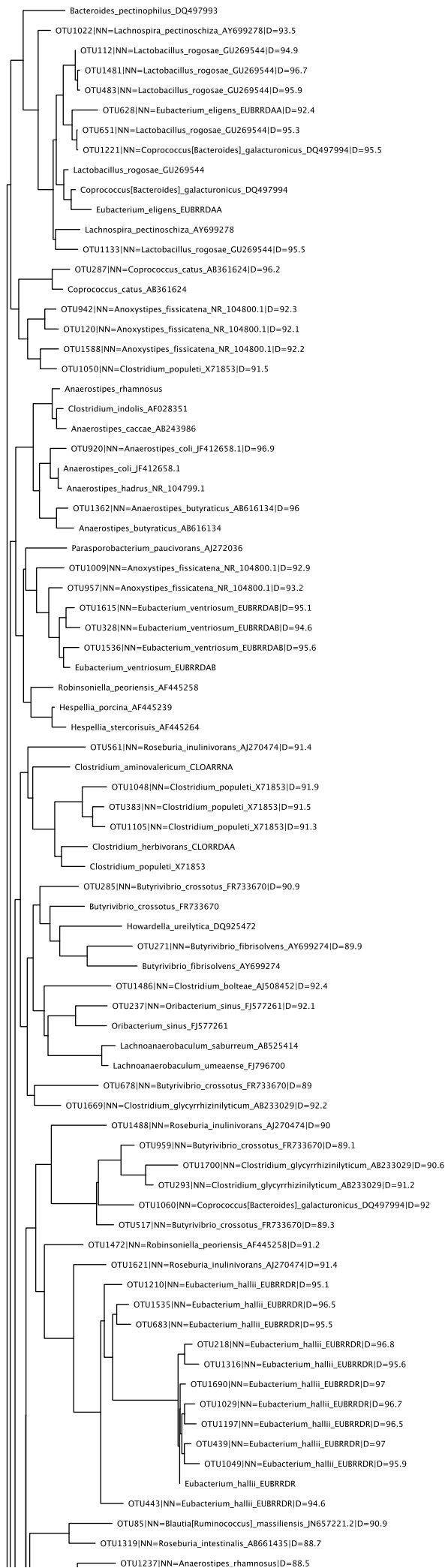

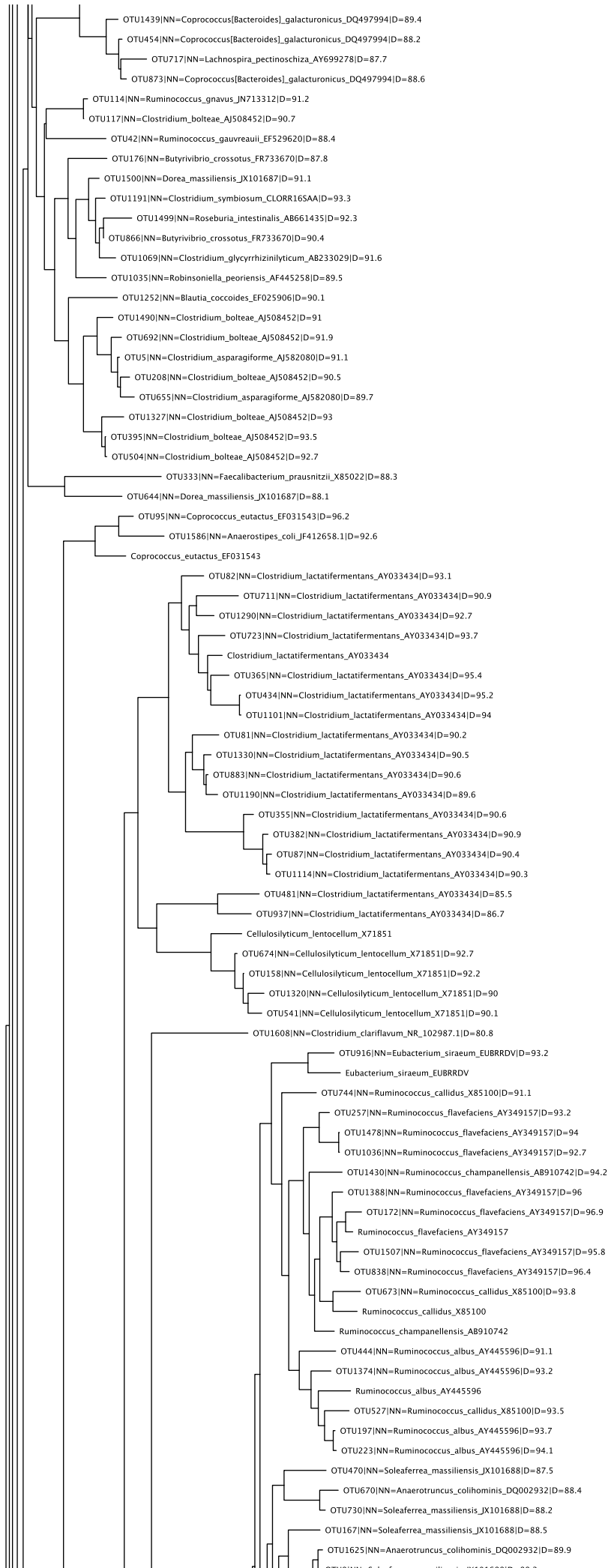

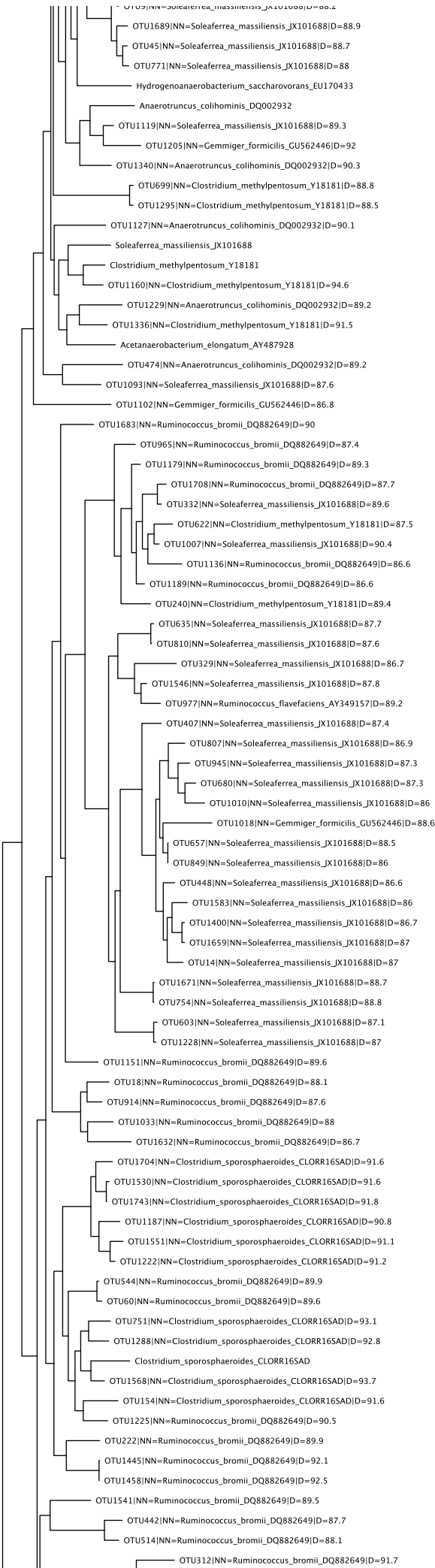

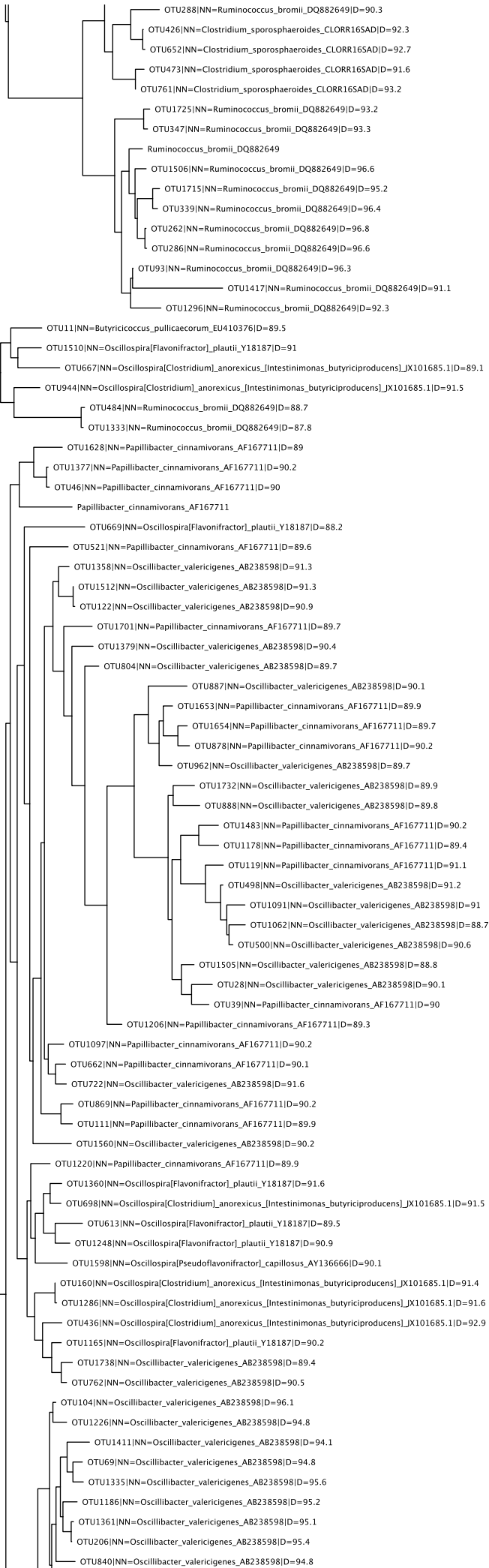

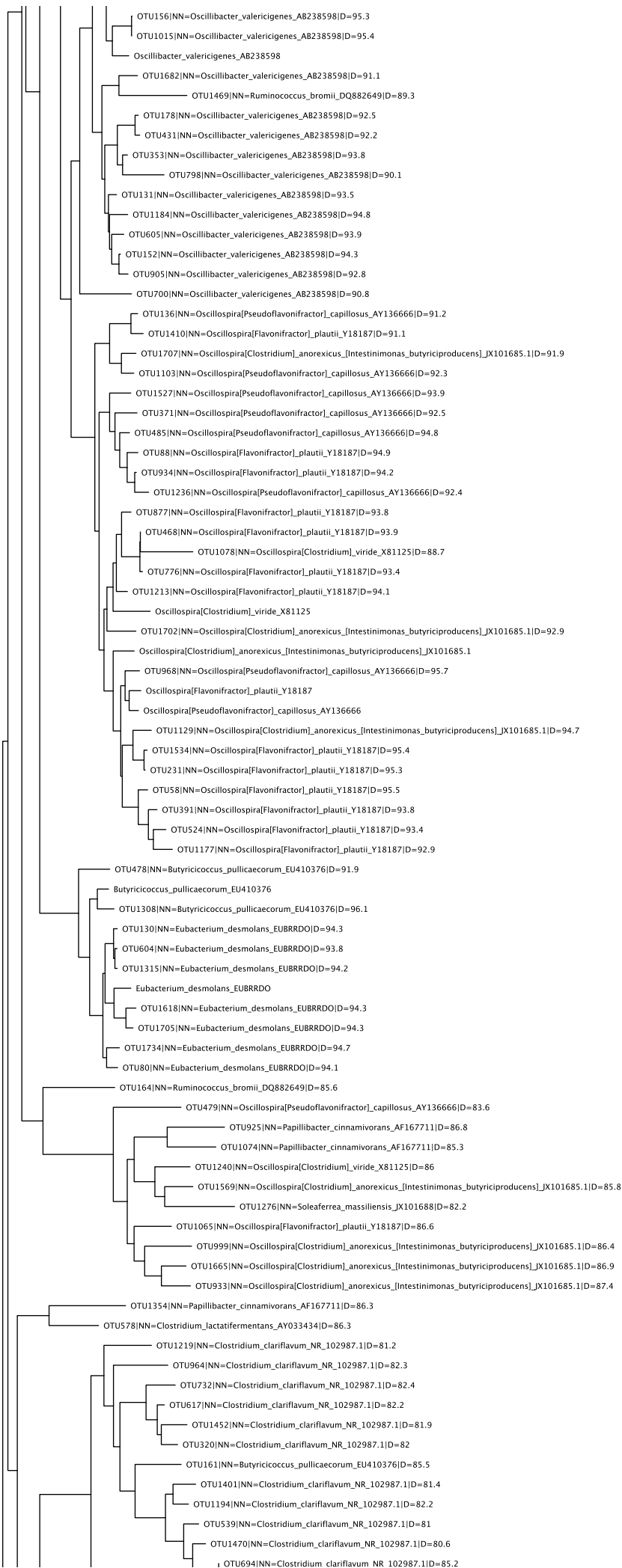

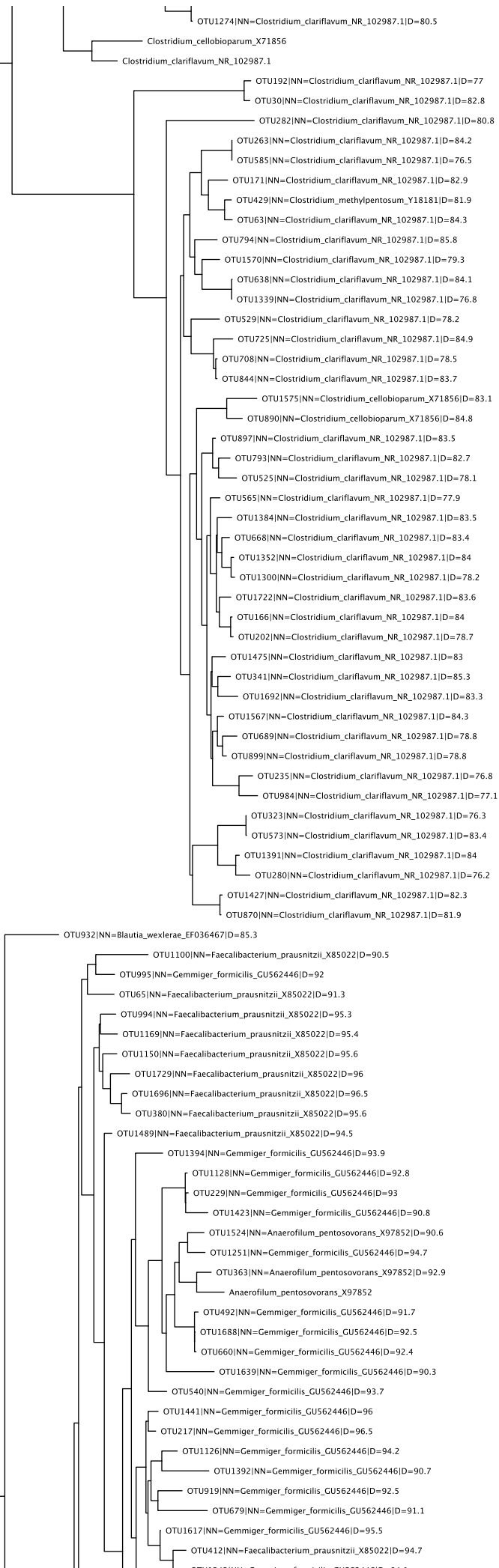

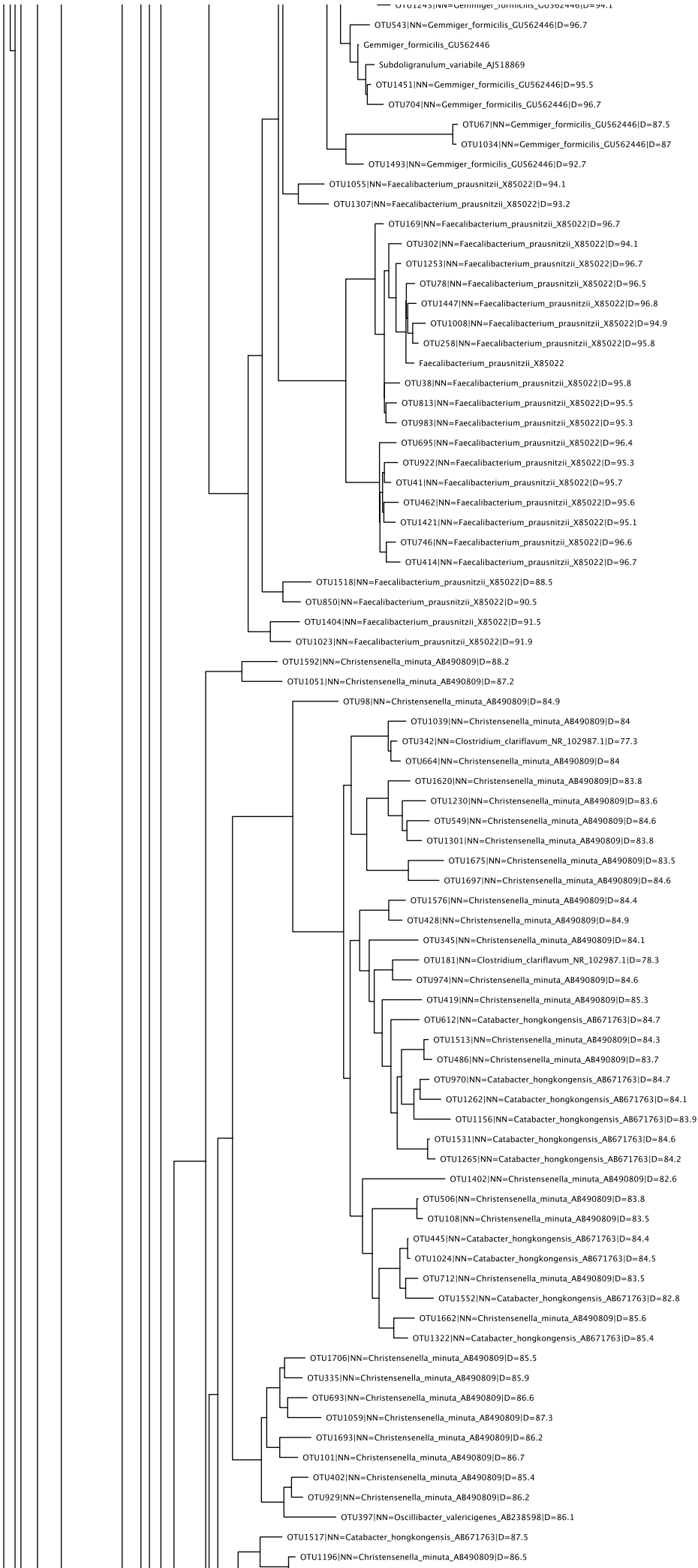

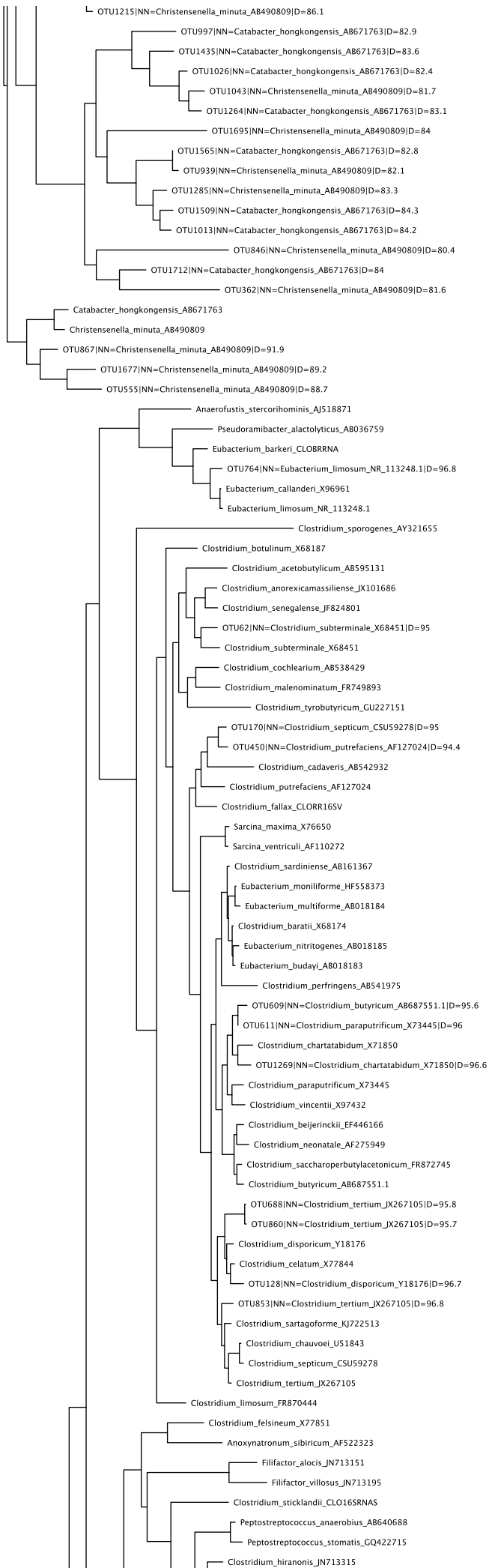

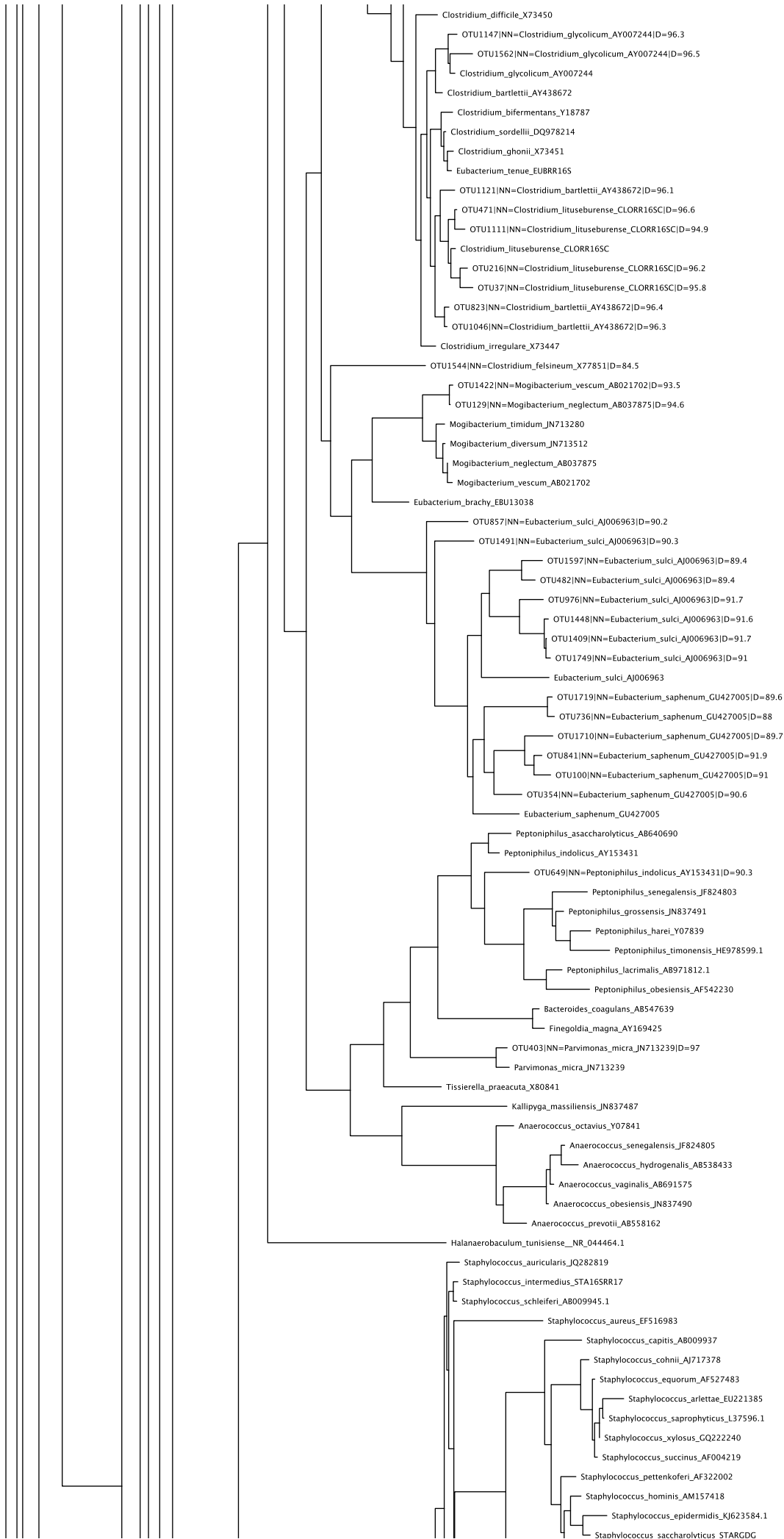

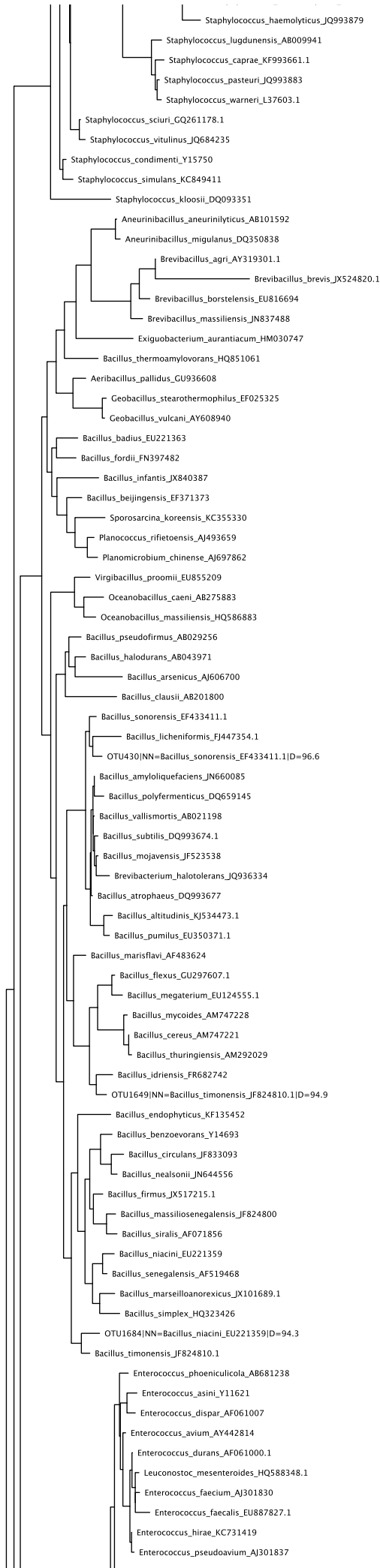

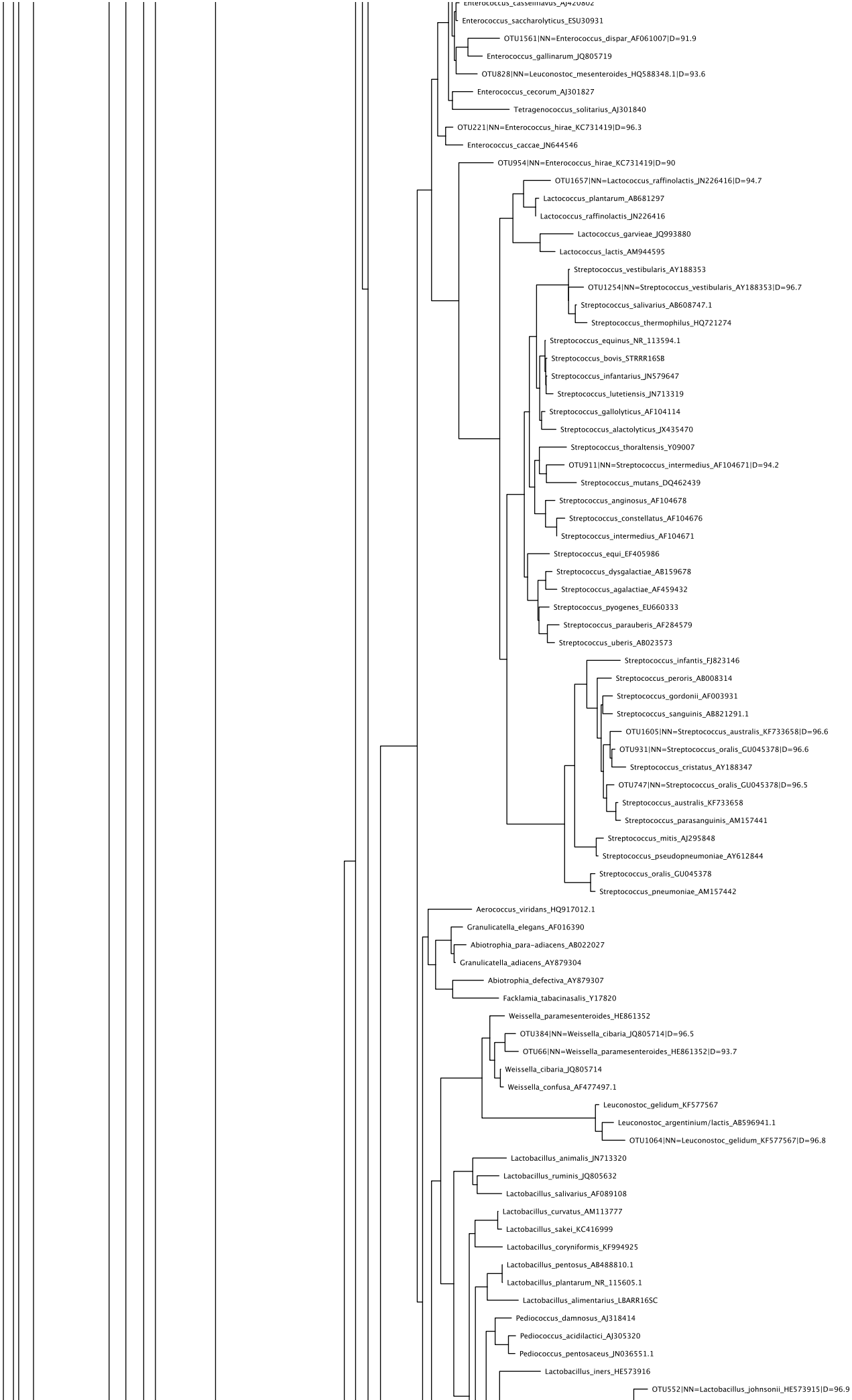

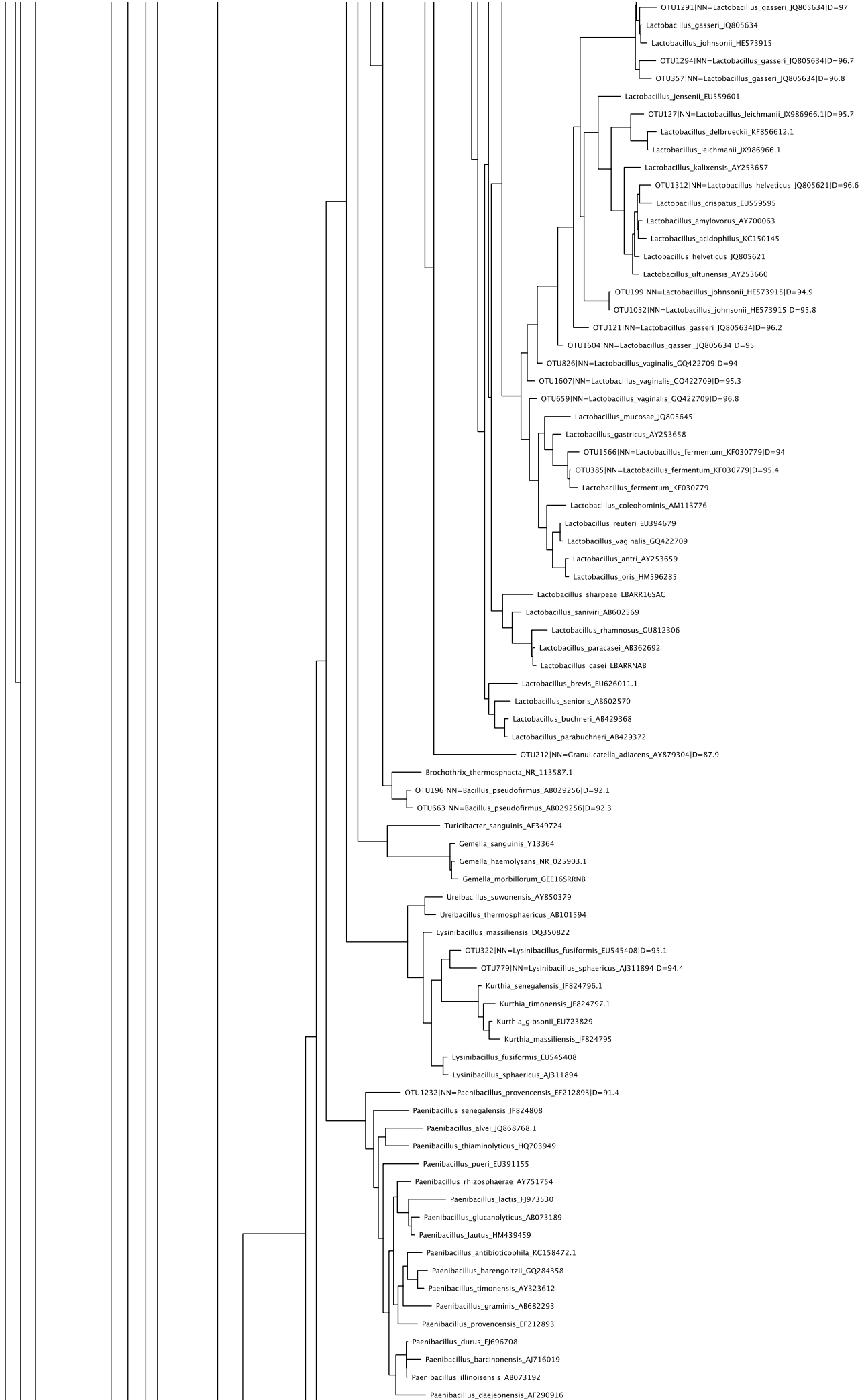

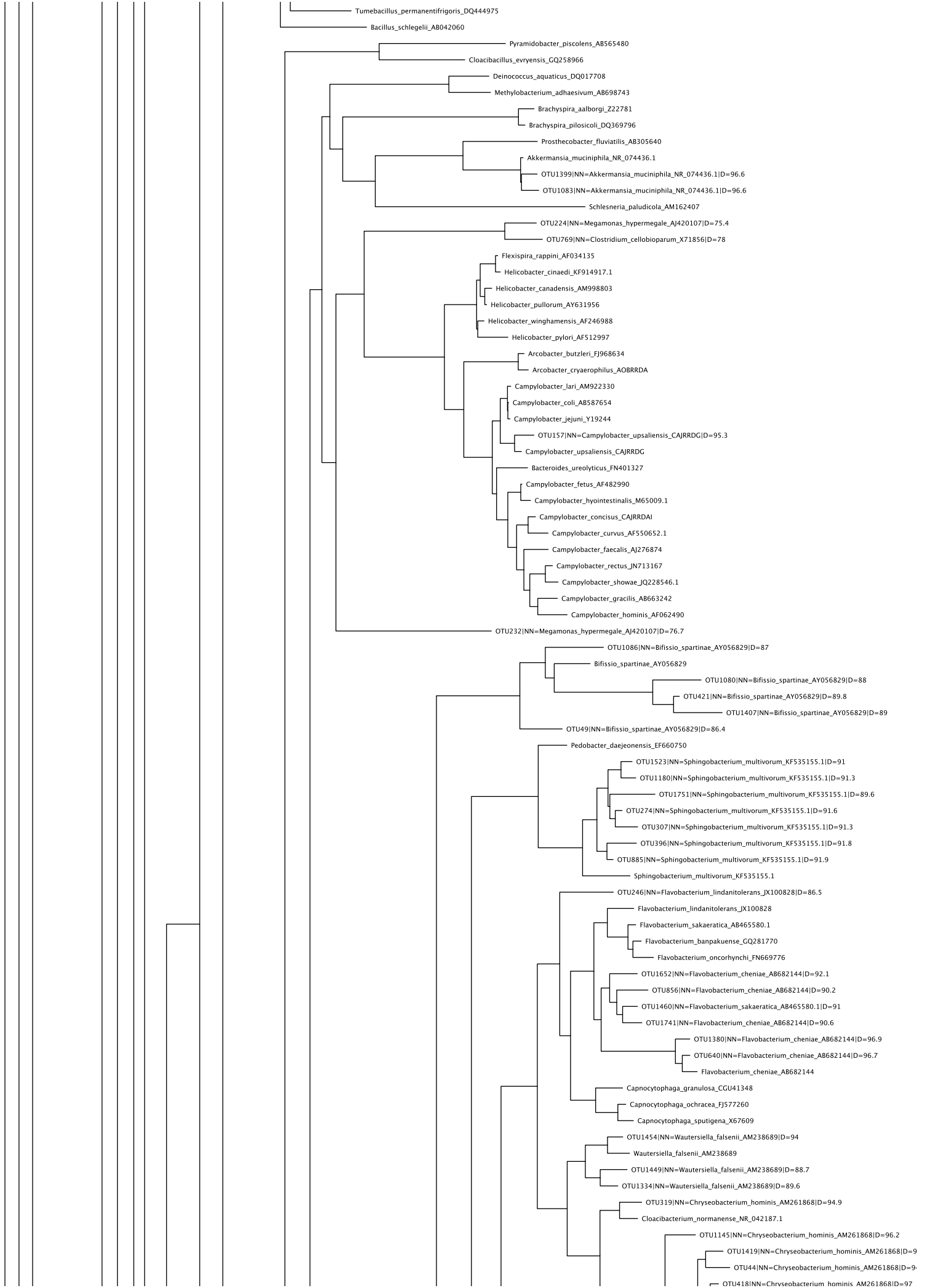

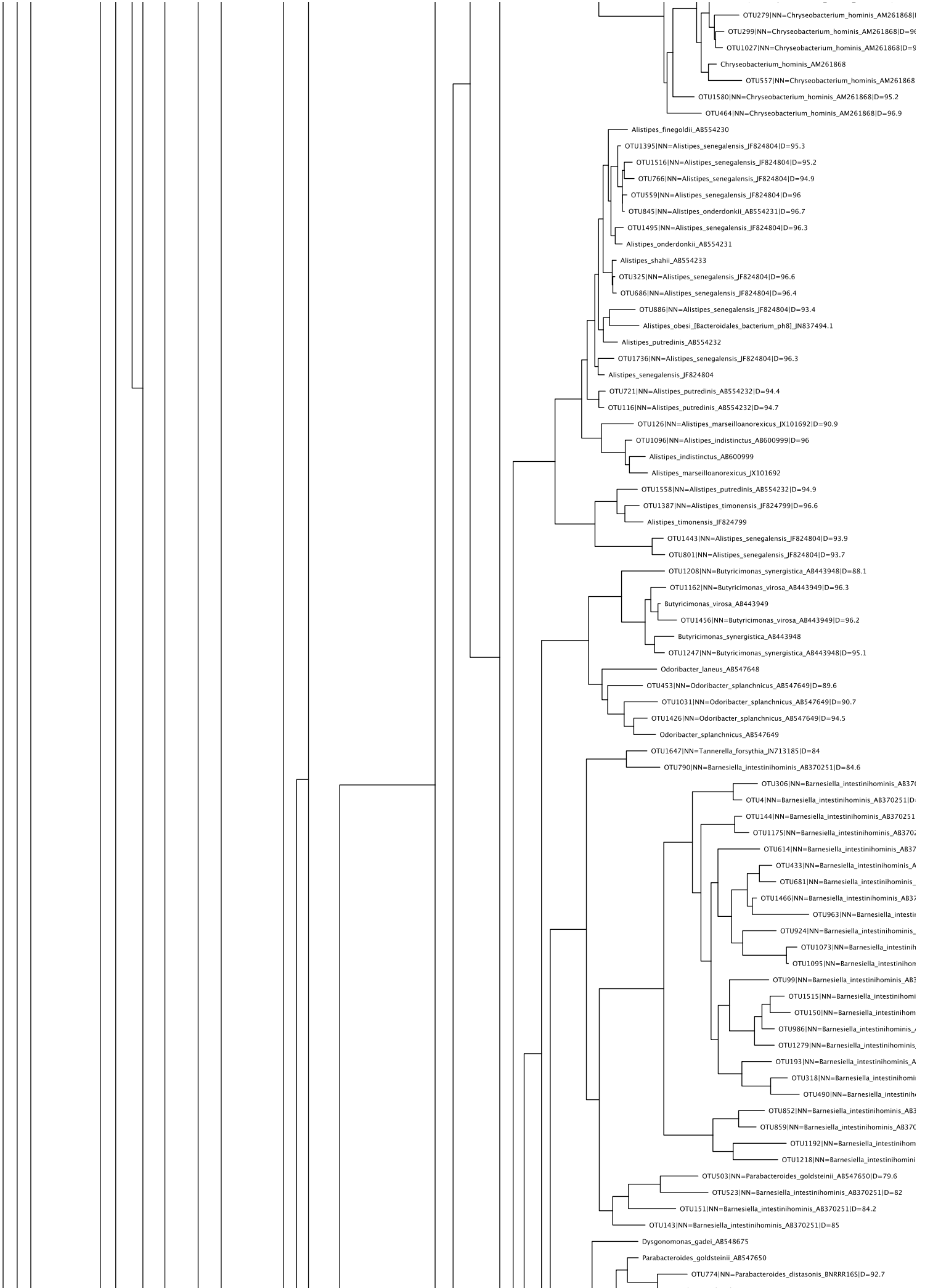

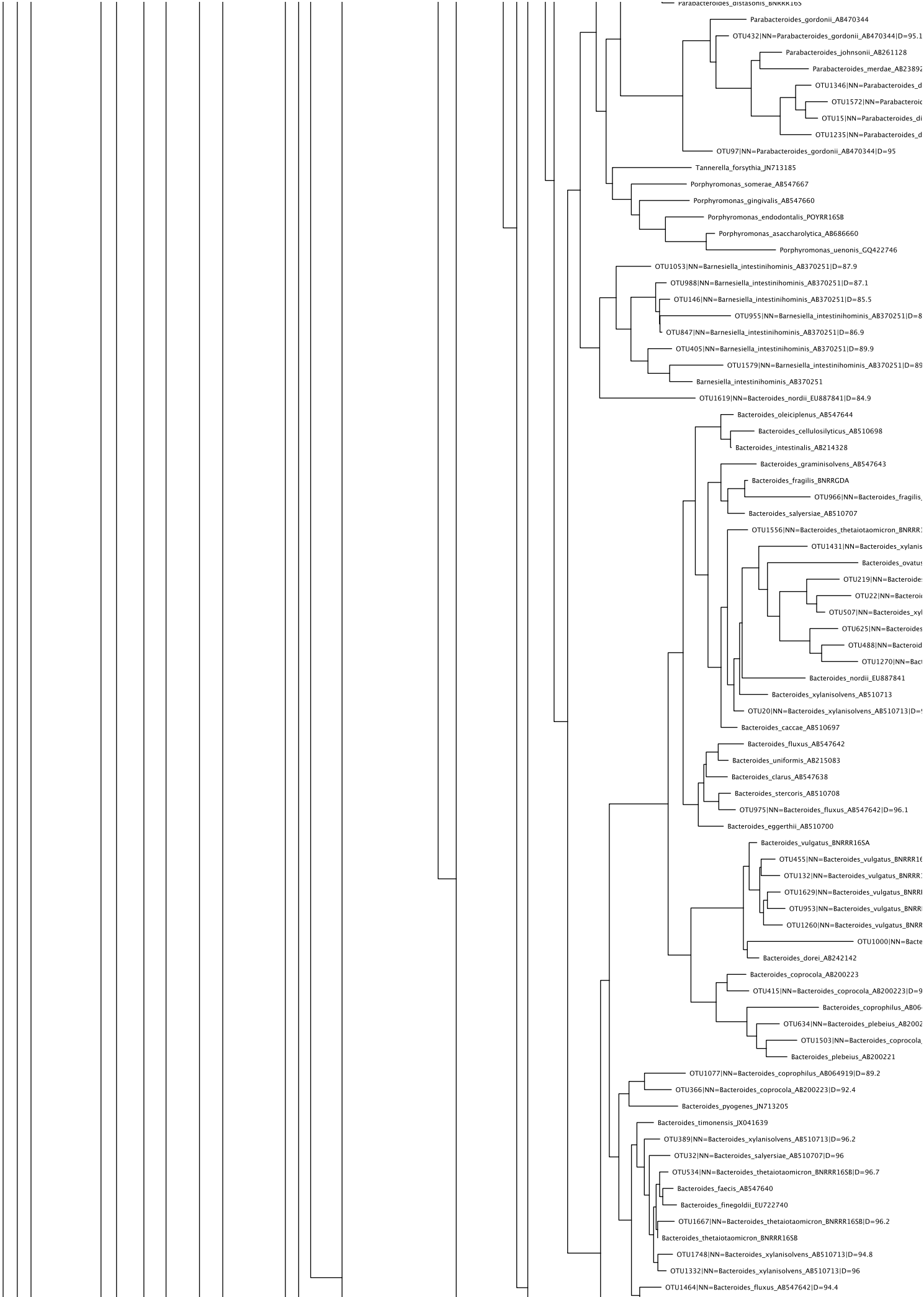

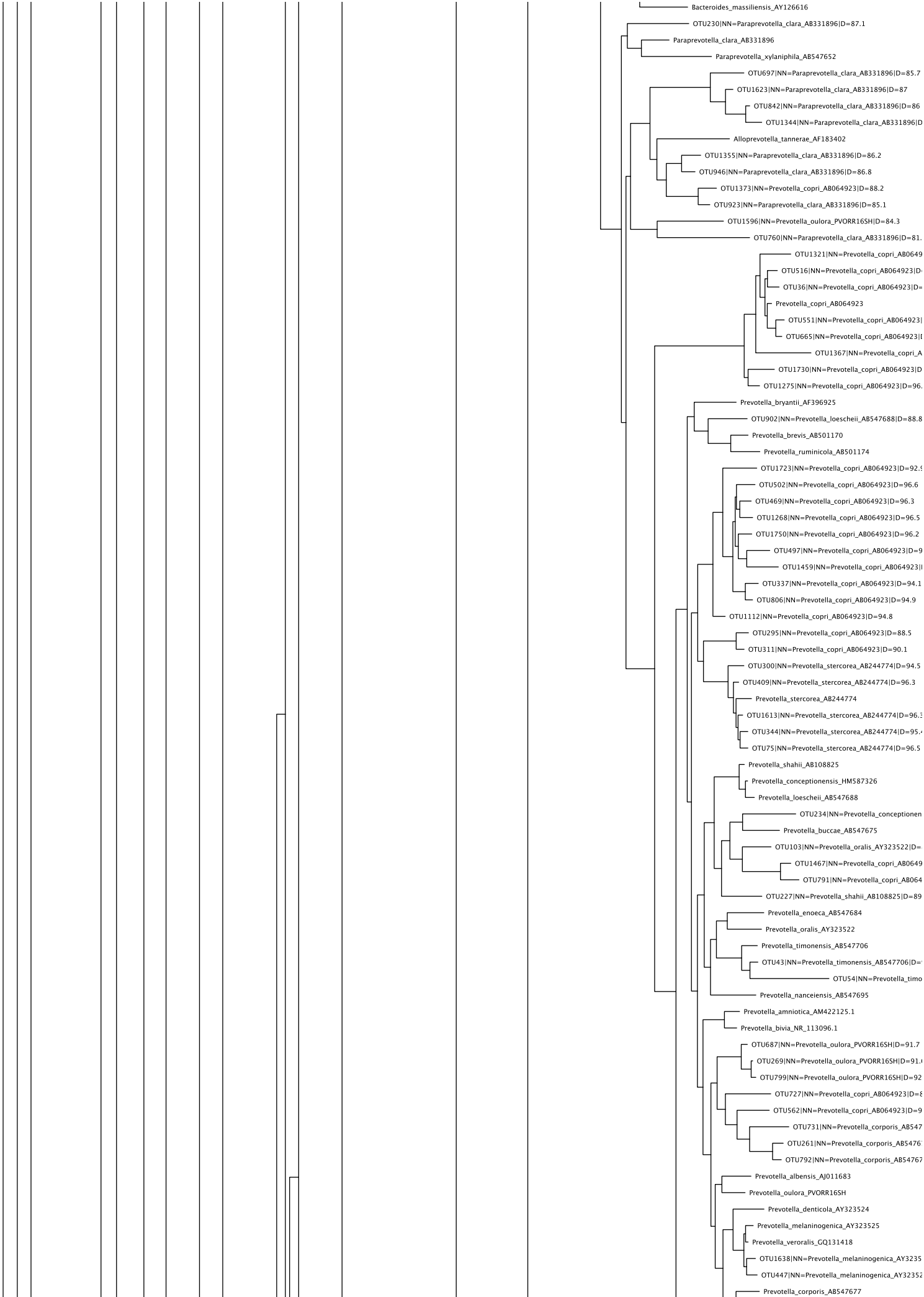

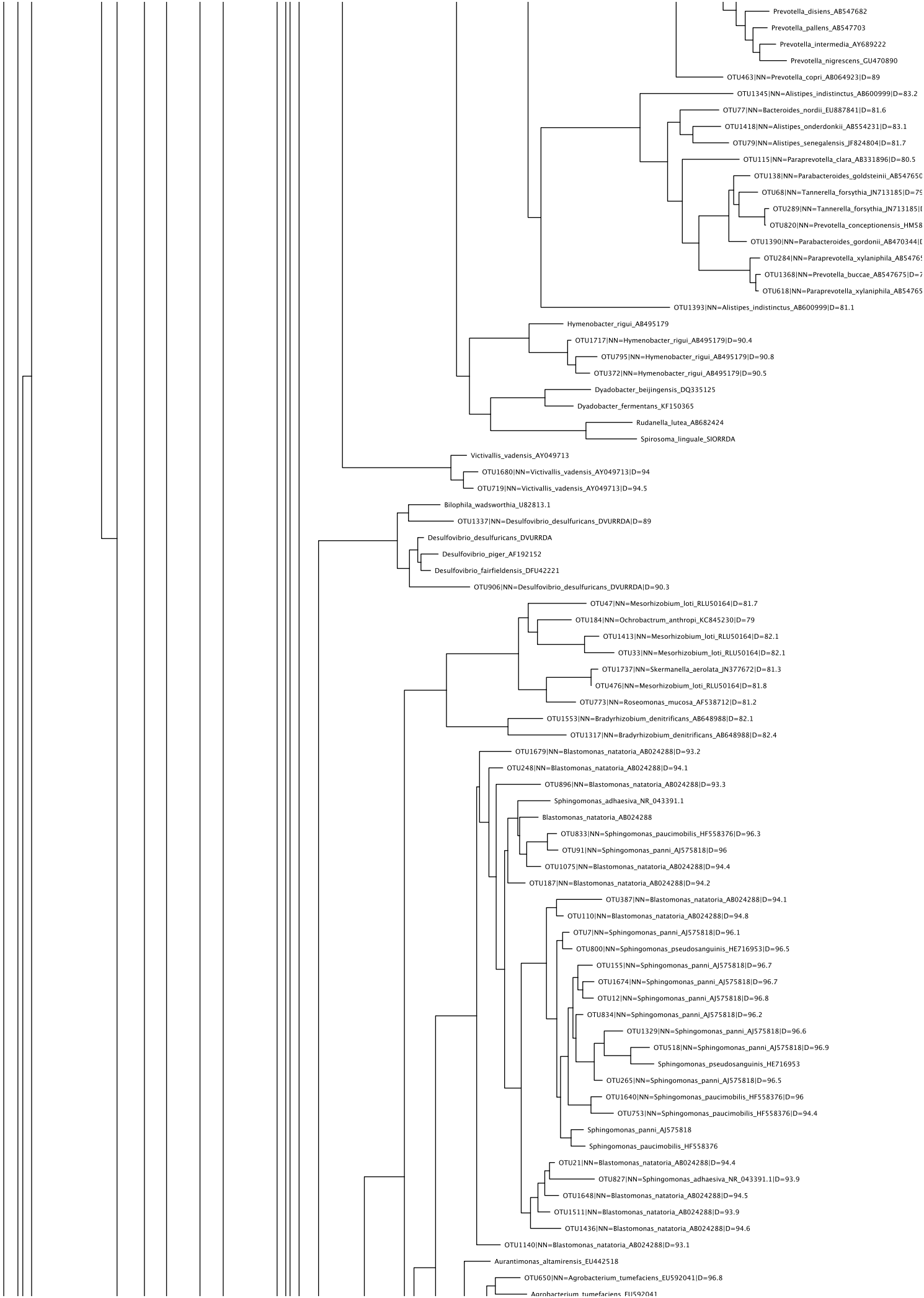

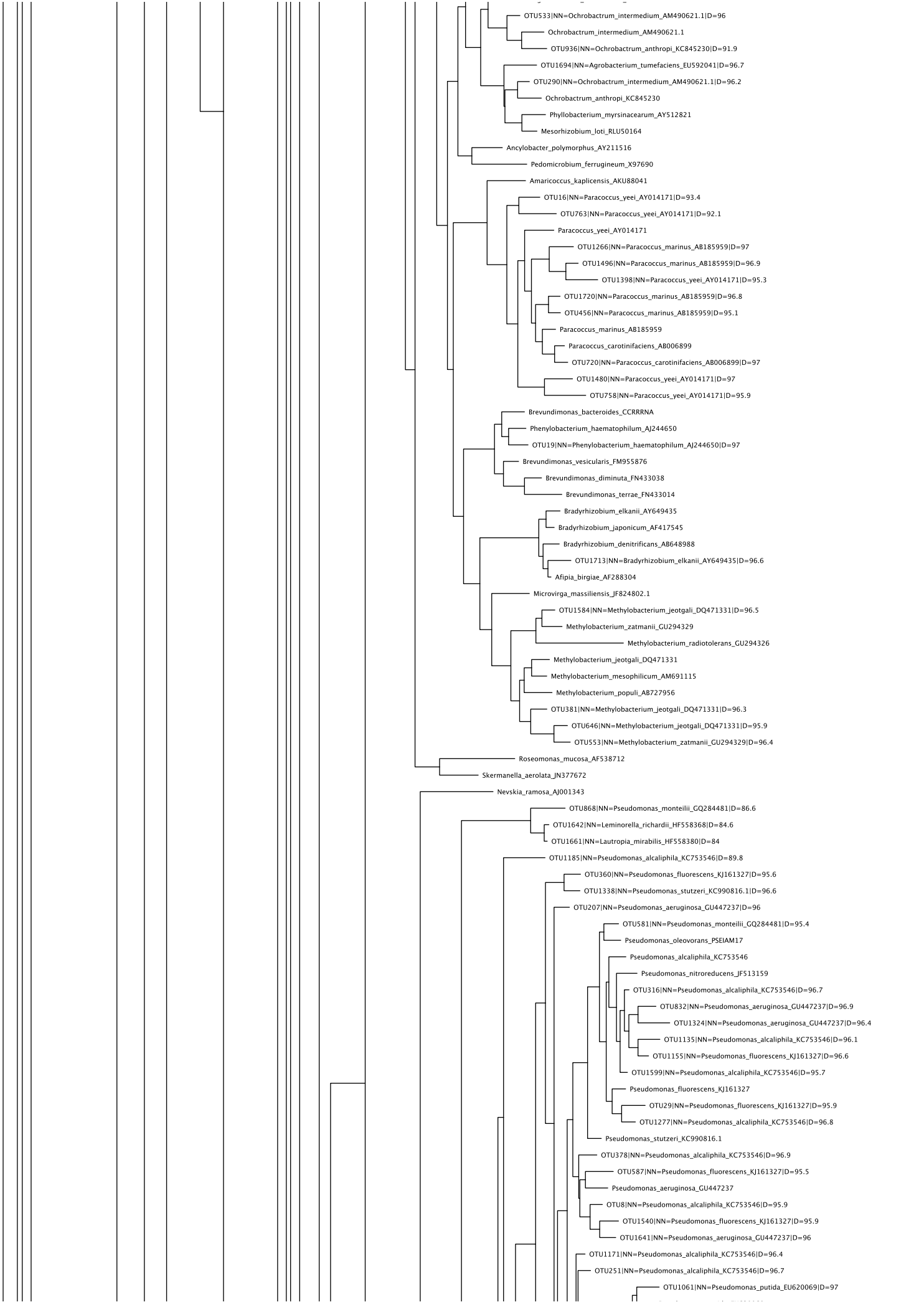

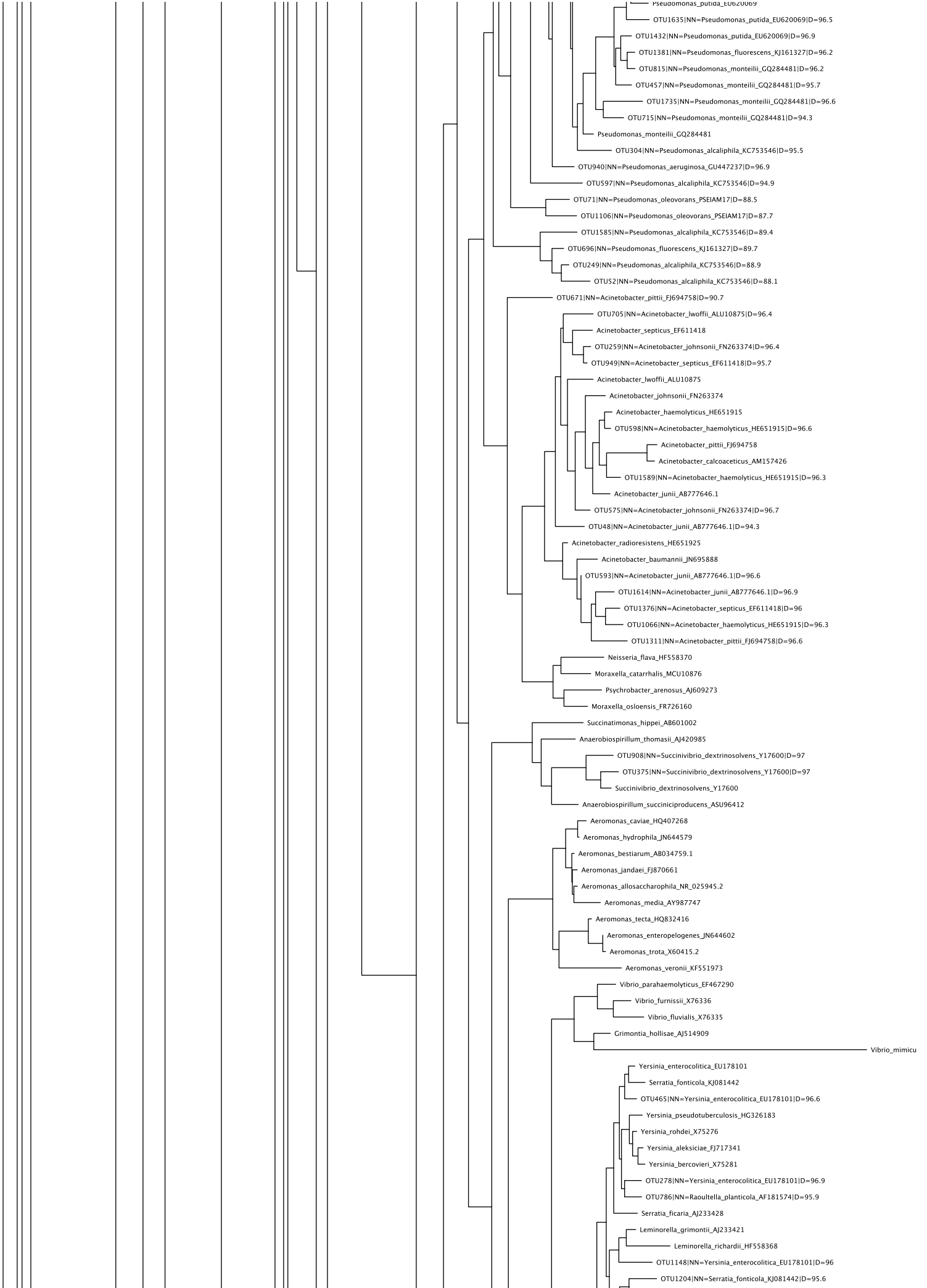

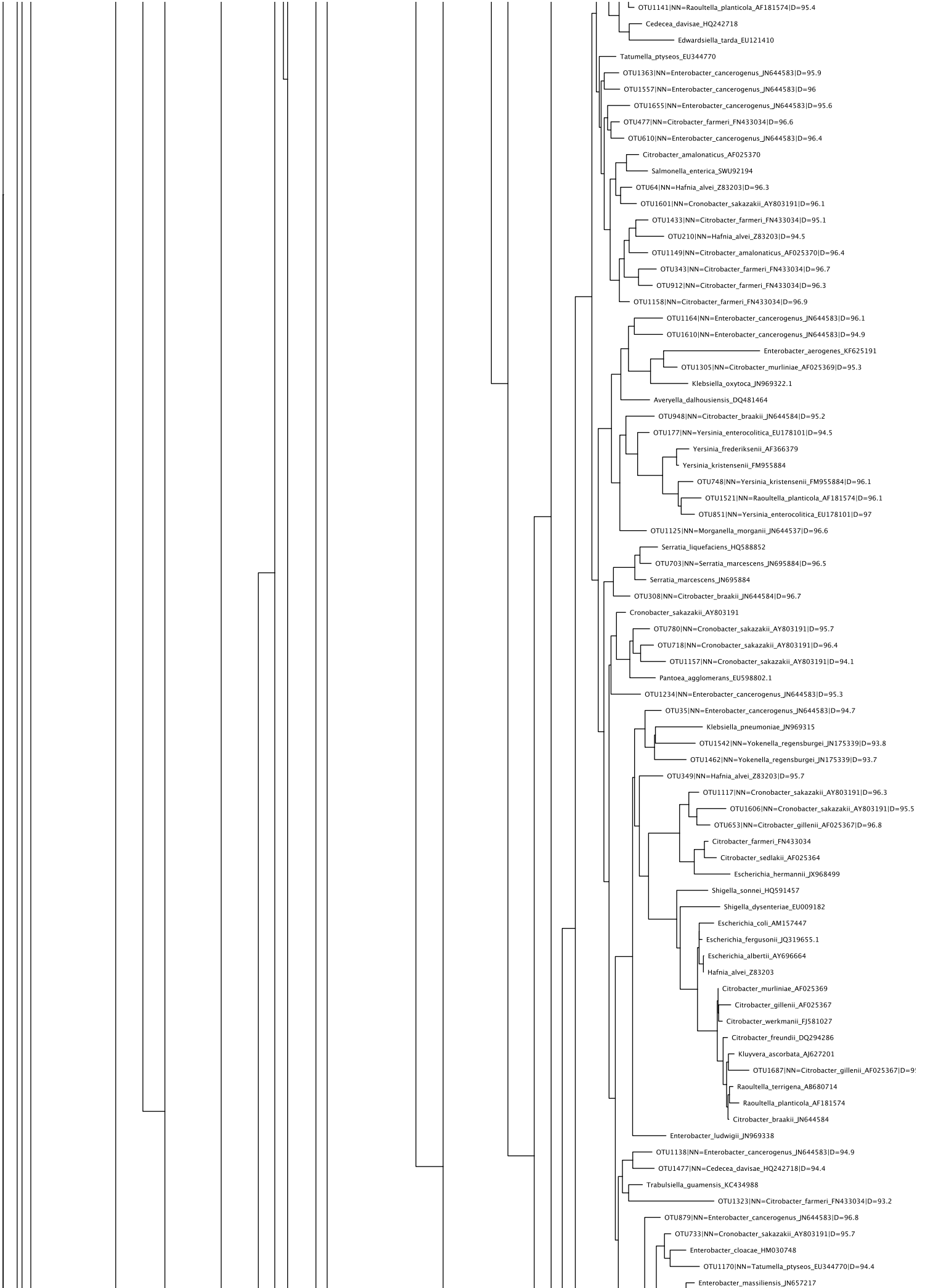

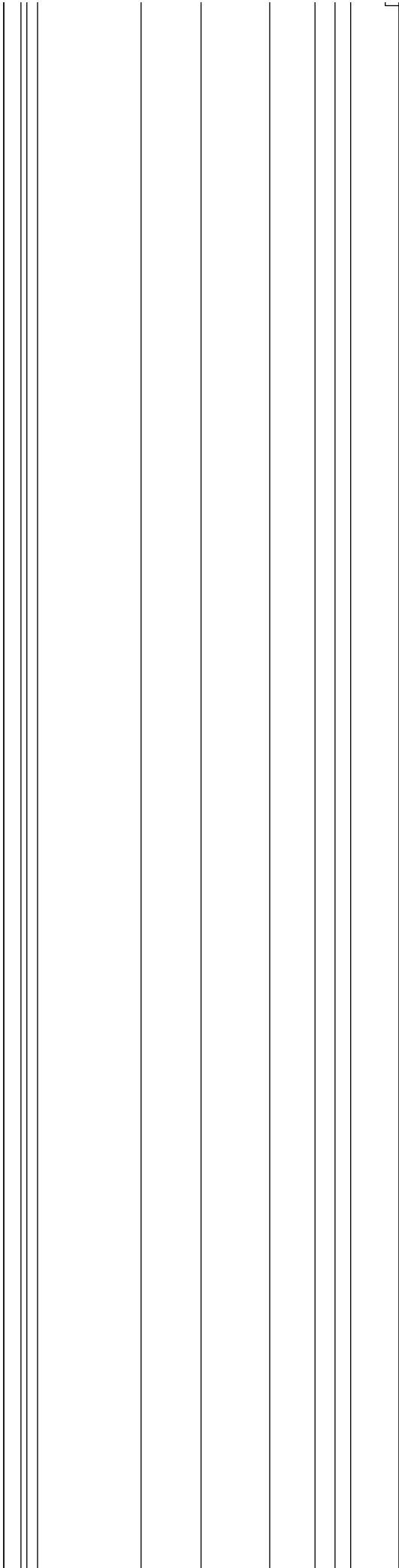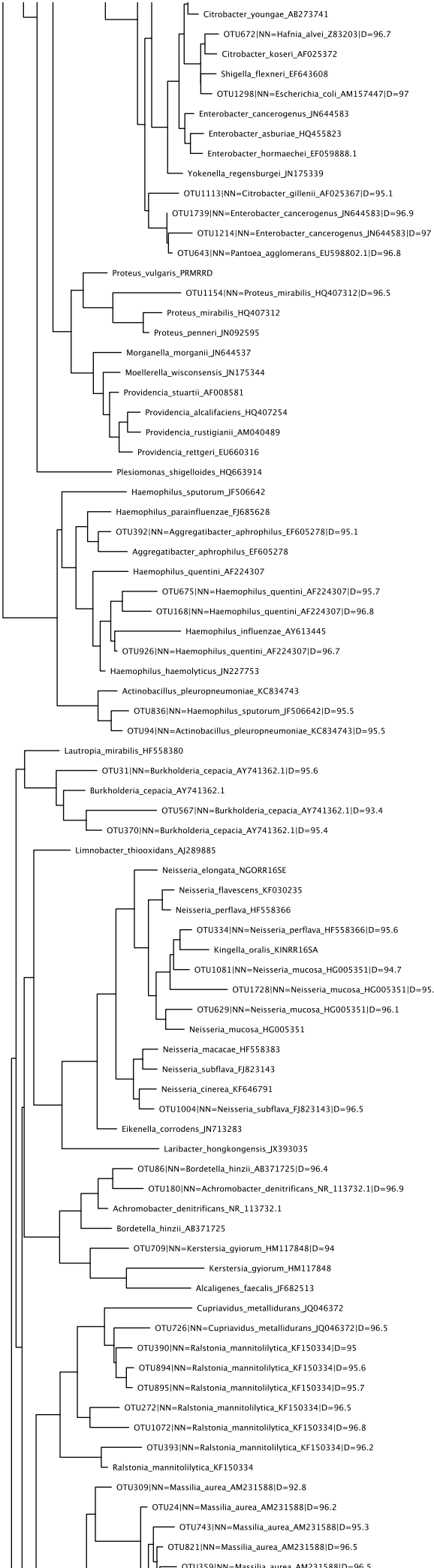

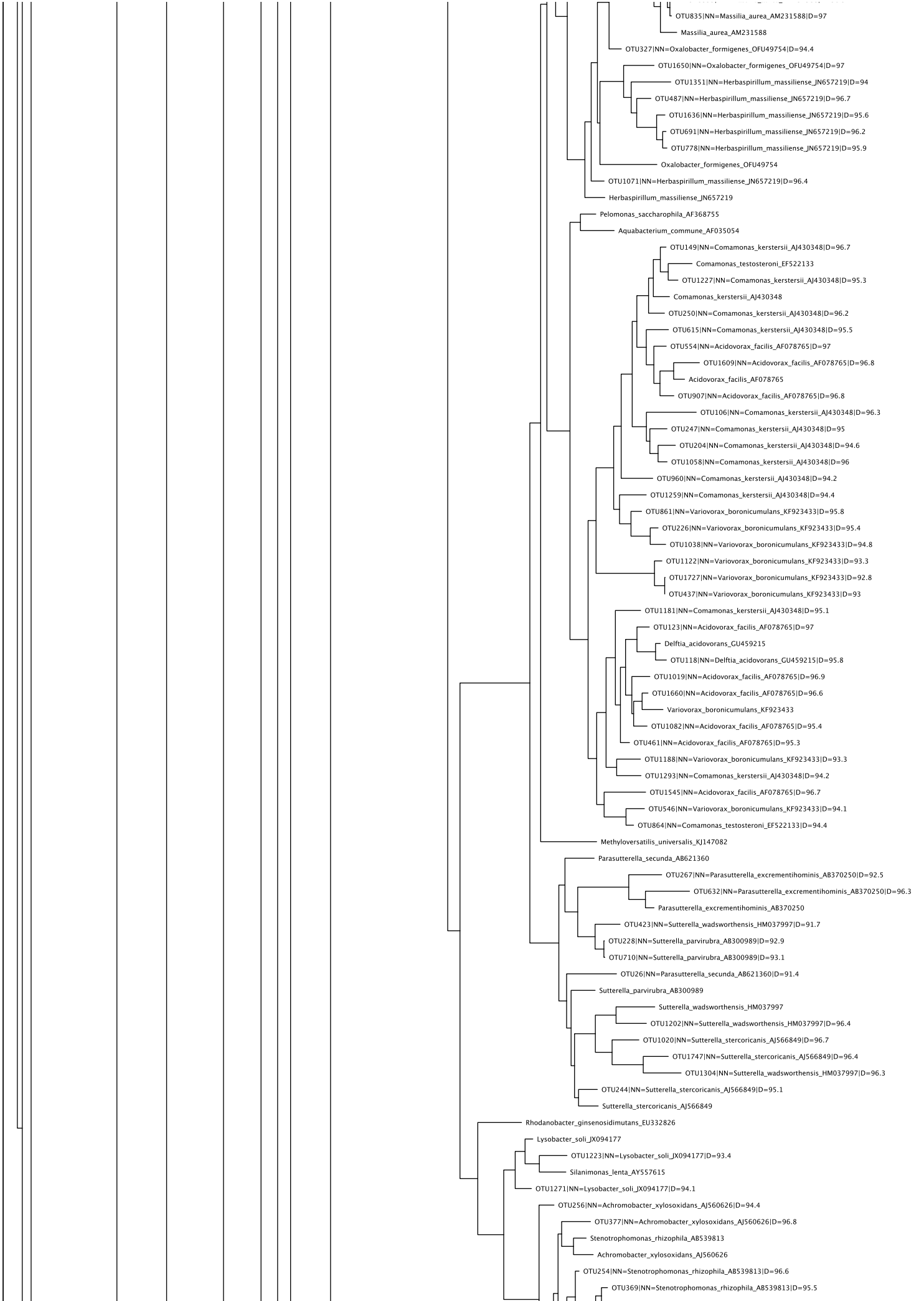

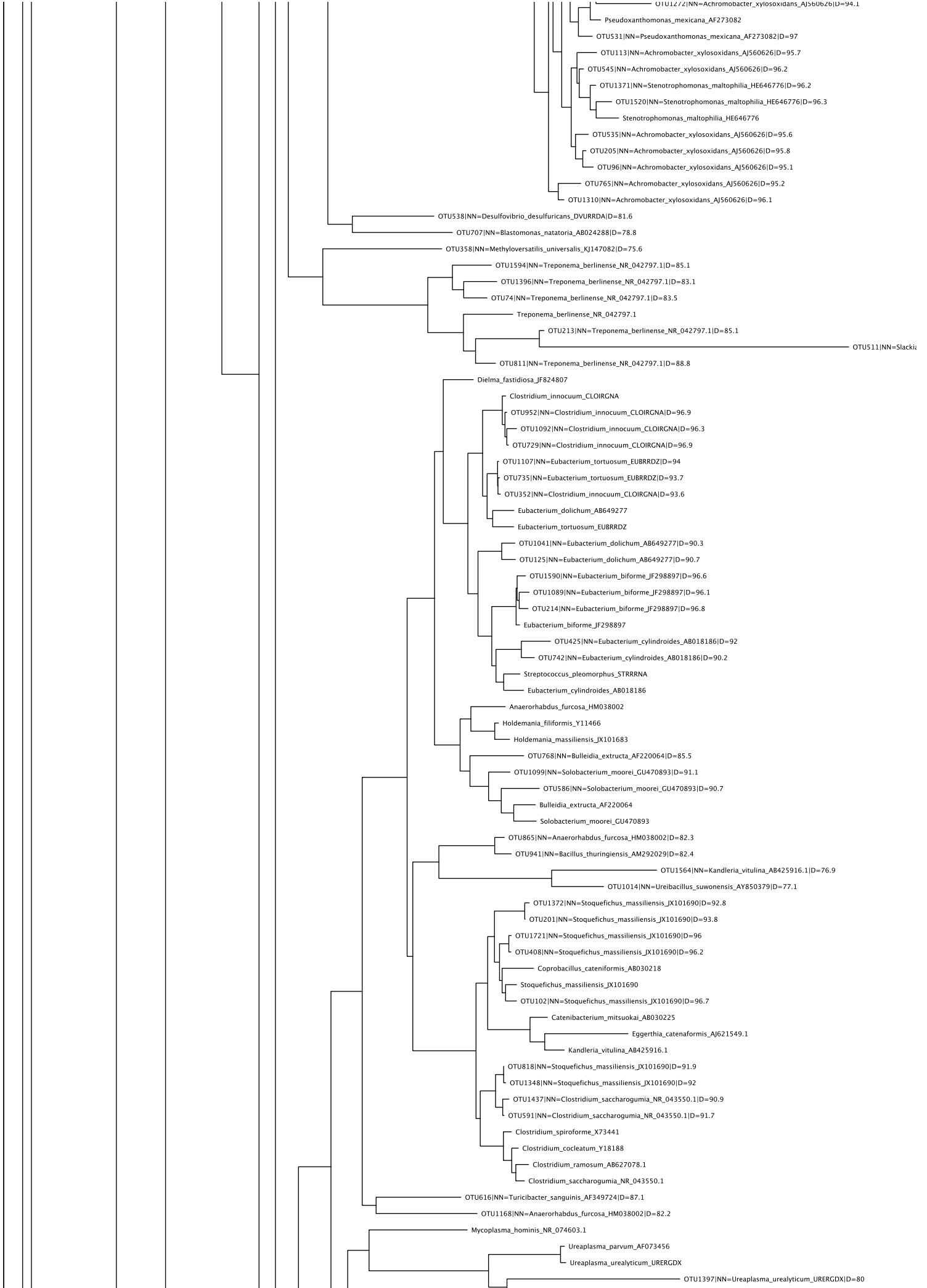

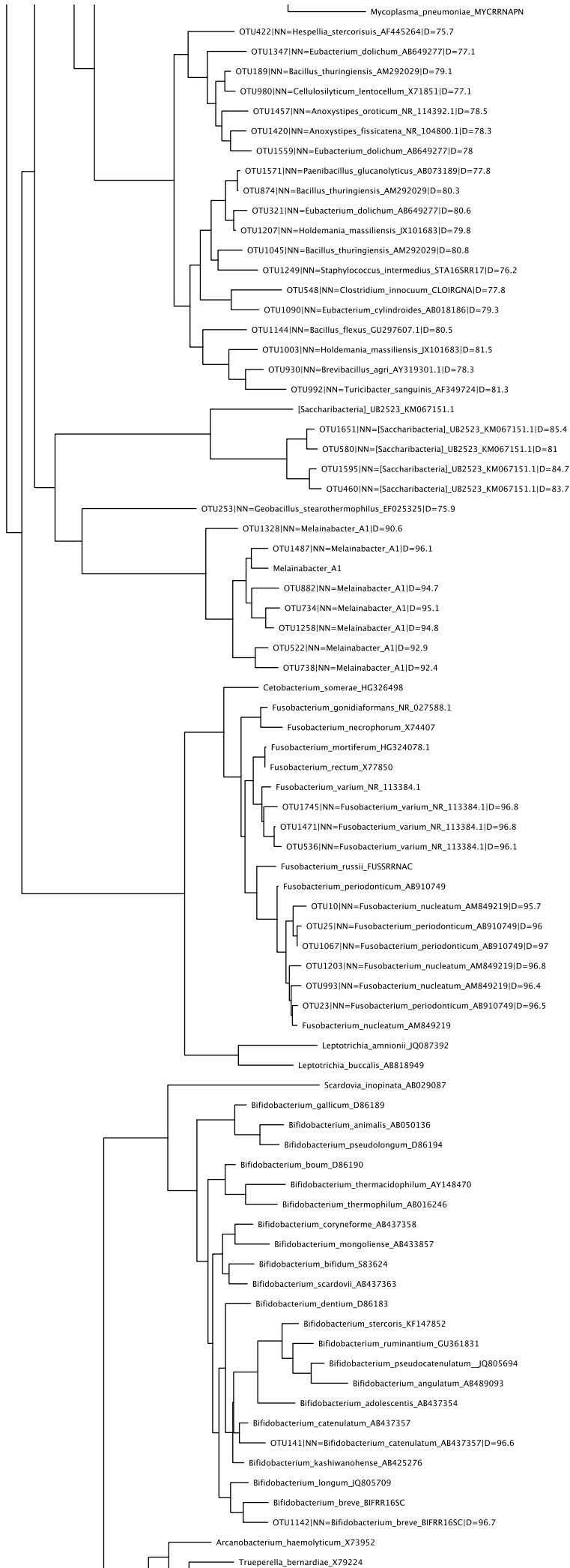

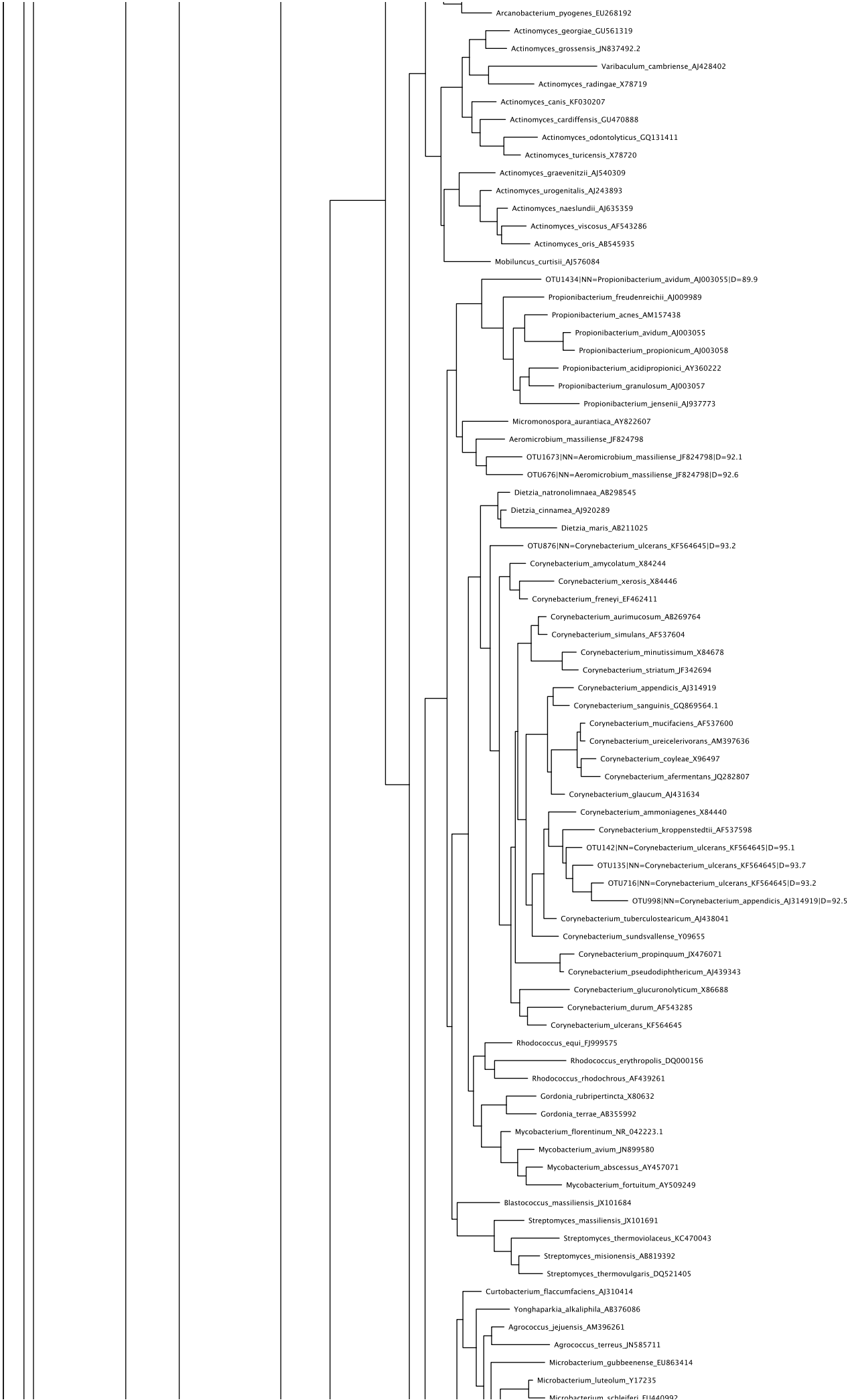

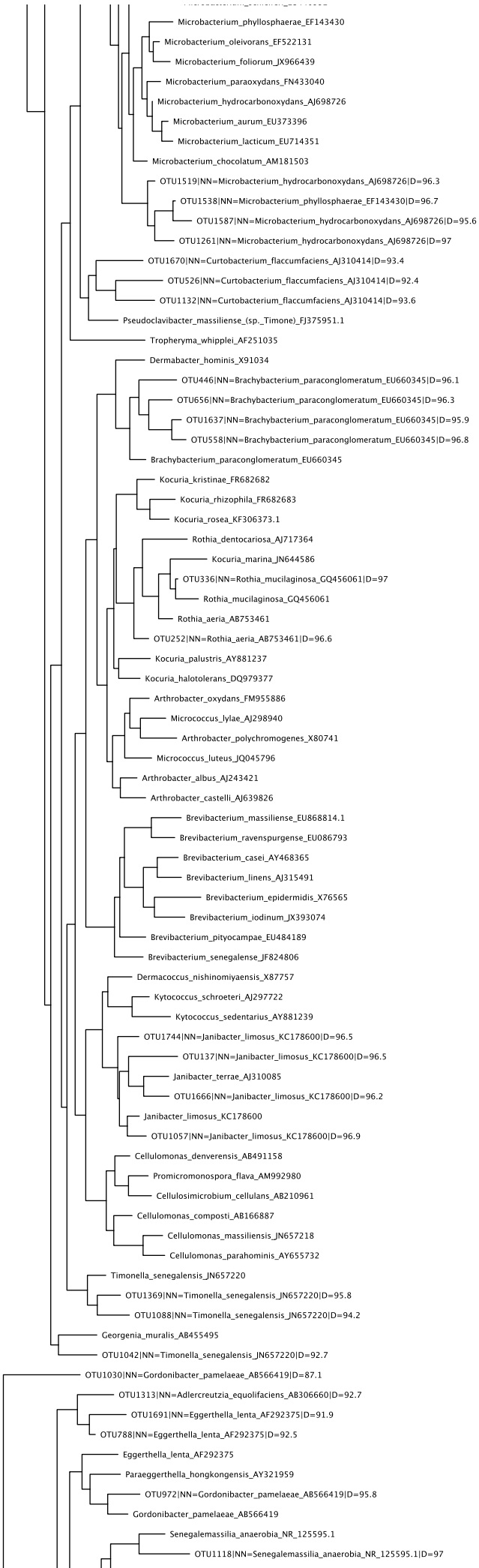

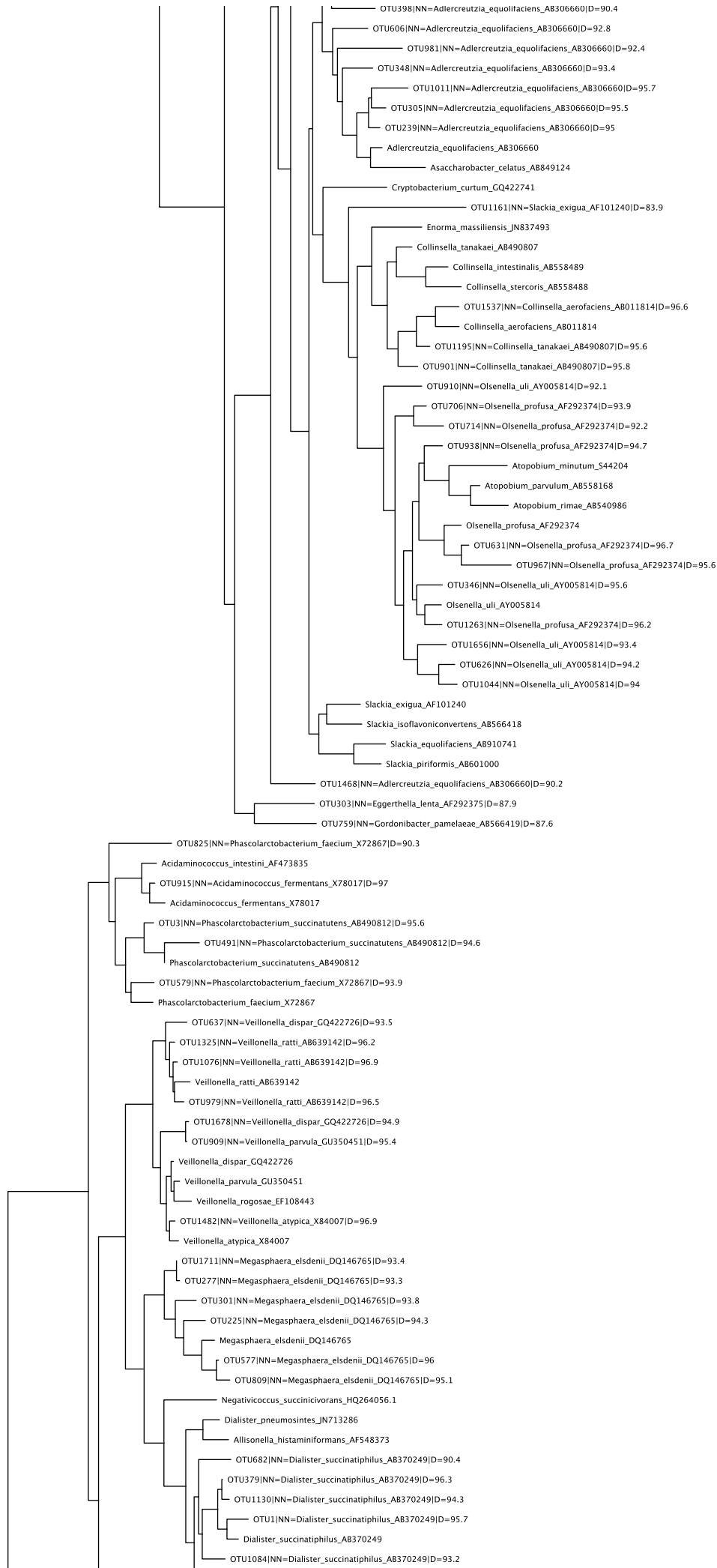

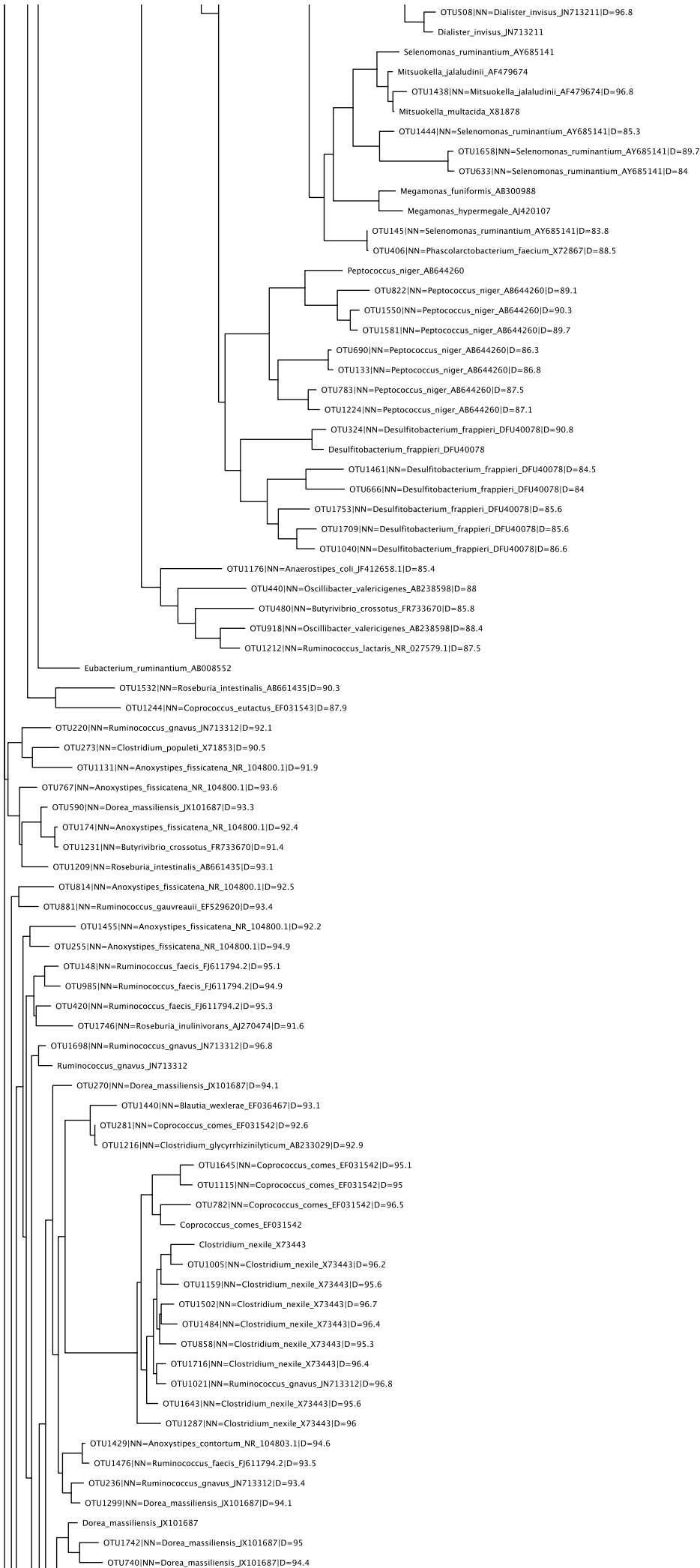

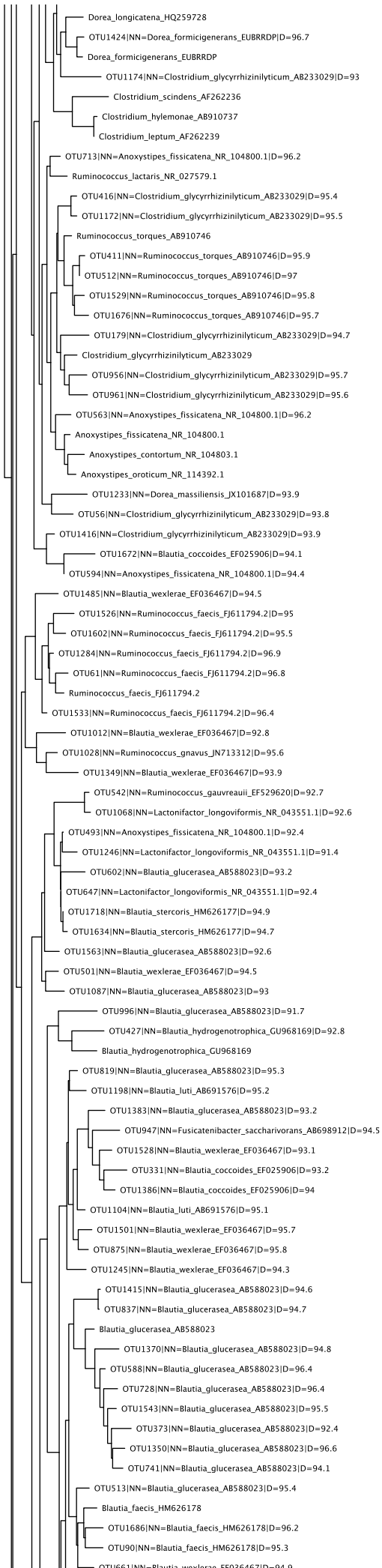

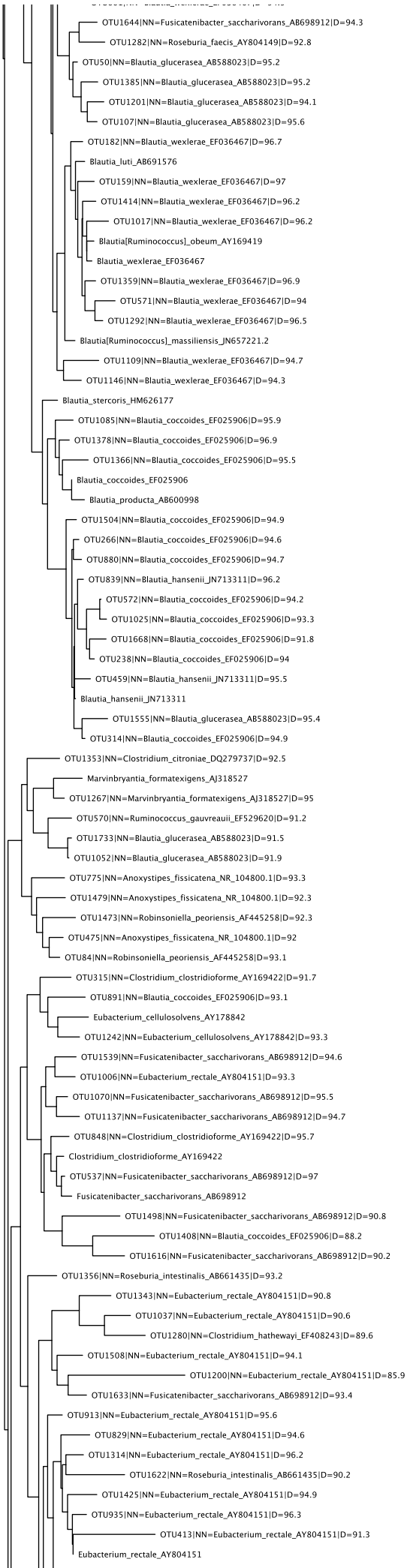

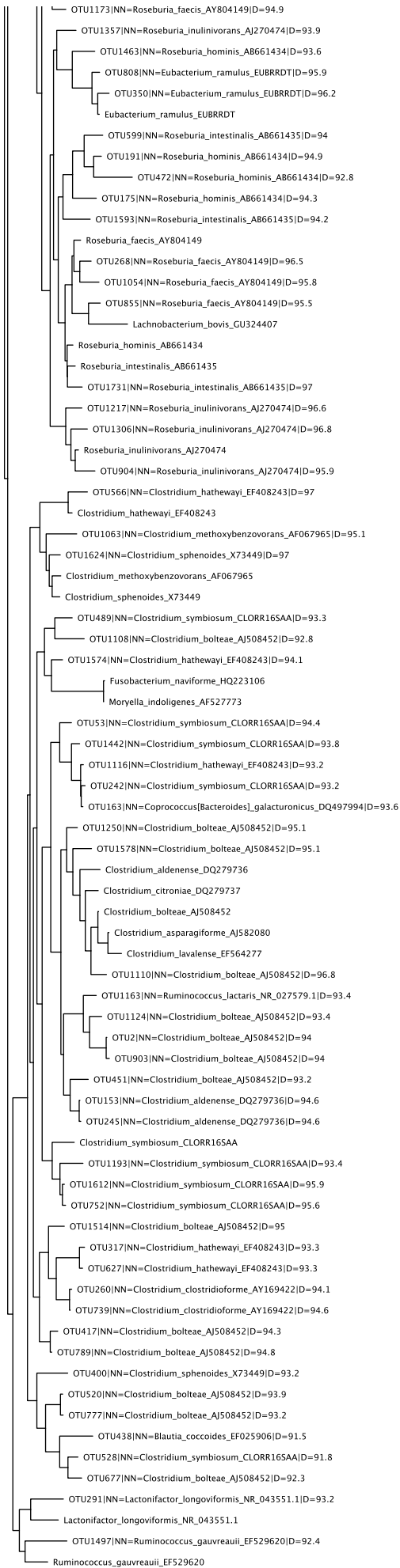

Supplement: Additional file 1: — Phylogenetic trees. Package containing Newick and figure files of bacterial and archaeal phylogenies in HITdb. (PDF 22521 kb) [file 12864_2015_2265_MOESM1_ESM.pdf]

# Computational rarefaction: Number of OTUs in samplings from multinomial distribution

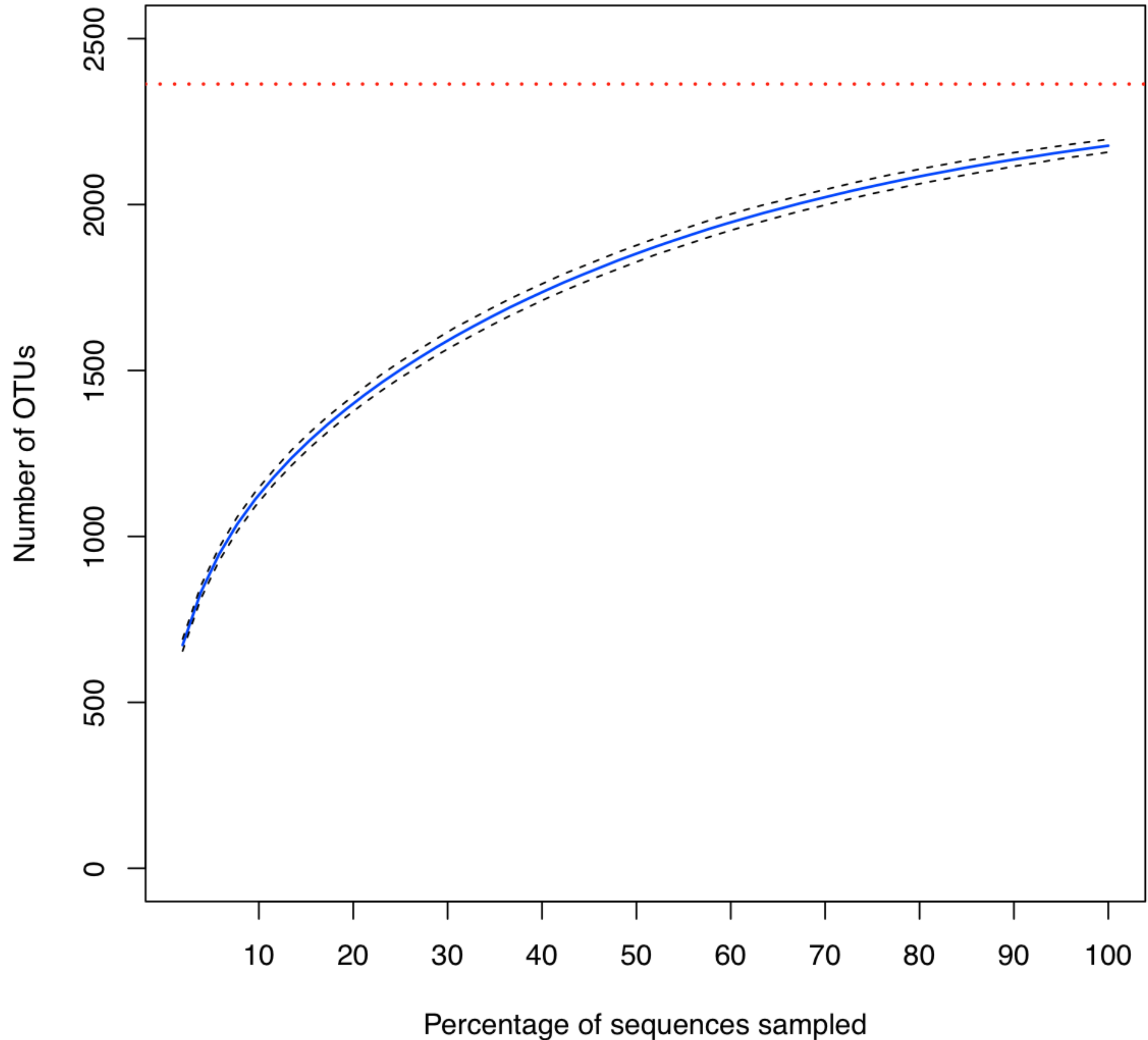

Supplement: Additional file 2: — Computational rarefaction. The figure shows the mean number of OTUs calculated from 5000 draws at different sample sizes from multinomial distribution. The dashed lines indicate the 0.95 and 0.05 quantiles. The horizontal red dotted line marks the number of all found clusters in the original data. (PDF 71 kb) [file 12864_2015_2265_MOESM2_ESM.pdf]

**A**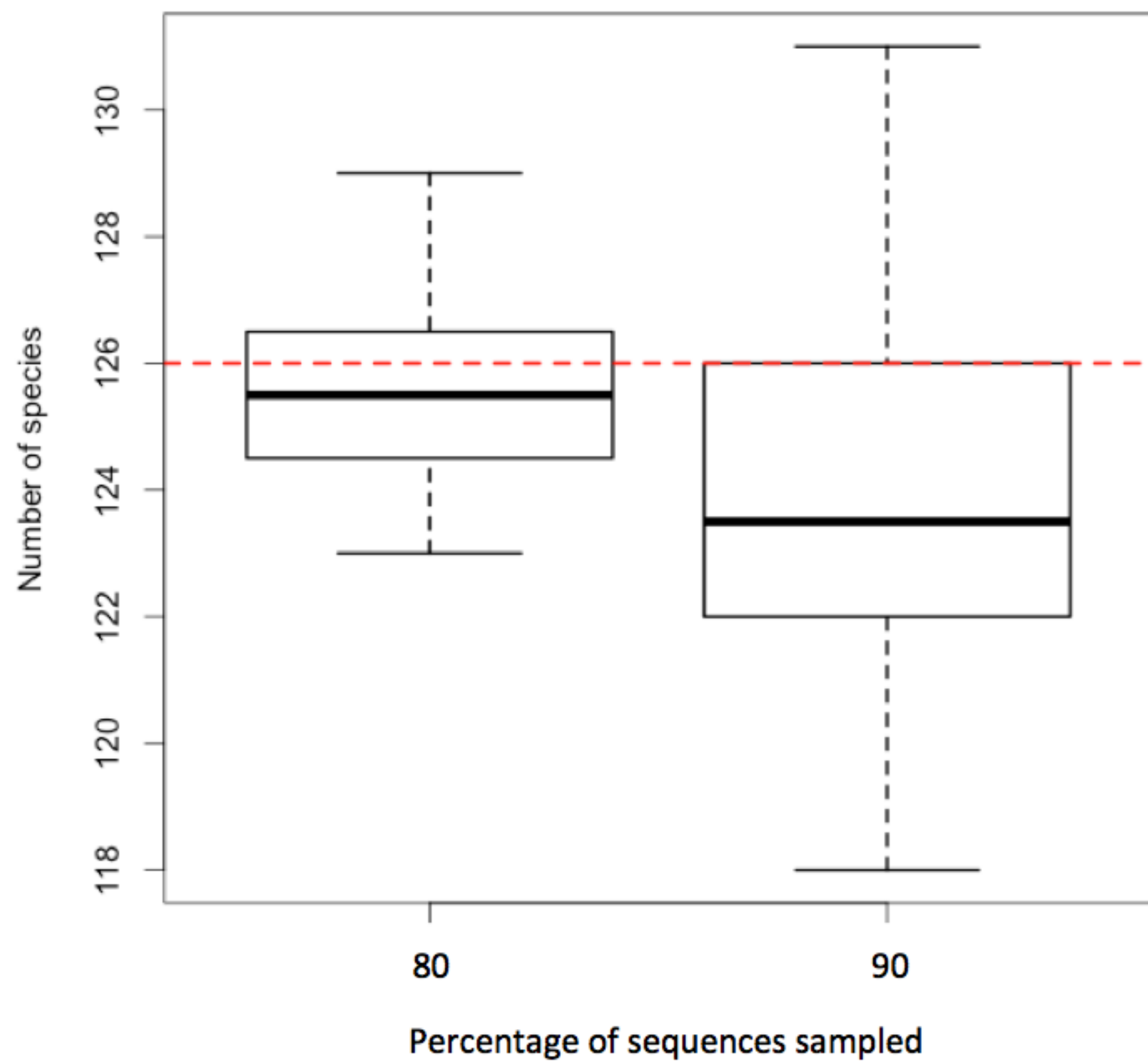**B**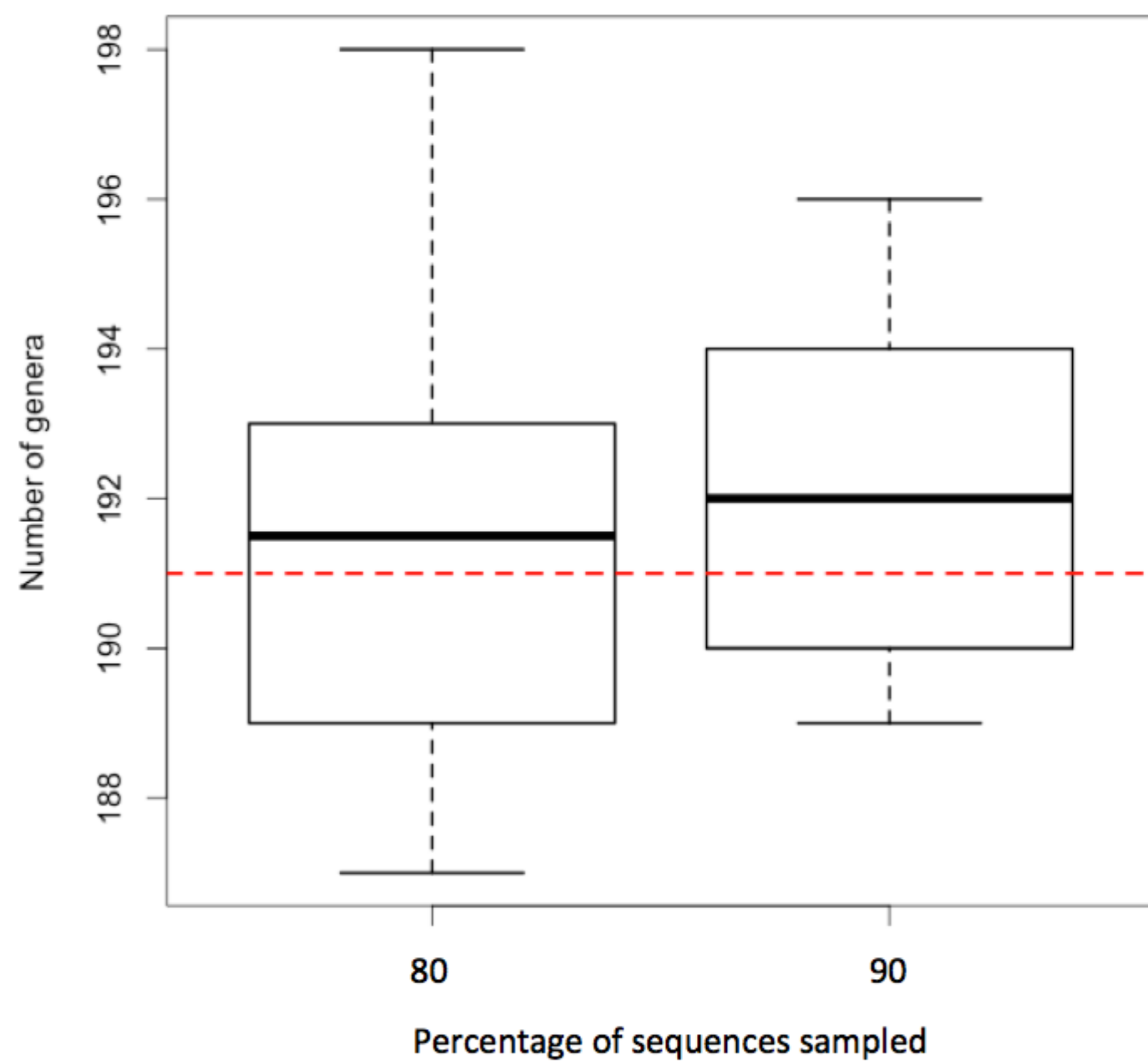

Supplement: Additional file 3: — Rarefaction based on known taxa. The figure shows the numbers of taxa calculated from samples of sequence data used for constructing the HITdb. Boxplots showing the numbers of found species (A) and genera (B) at two sample sizes (about 90 % and 80 % of sequences, n = 9 and n = 10, respectively). The horizontal red dashed line shows the number of OTUs in all sequences (100 % of sequences). (PDF 52 kb) [file 12864_2015_2265_MOESM3_ESM.pdf]

# Relative number of assigned taxa in synthetic reads

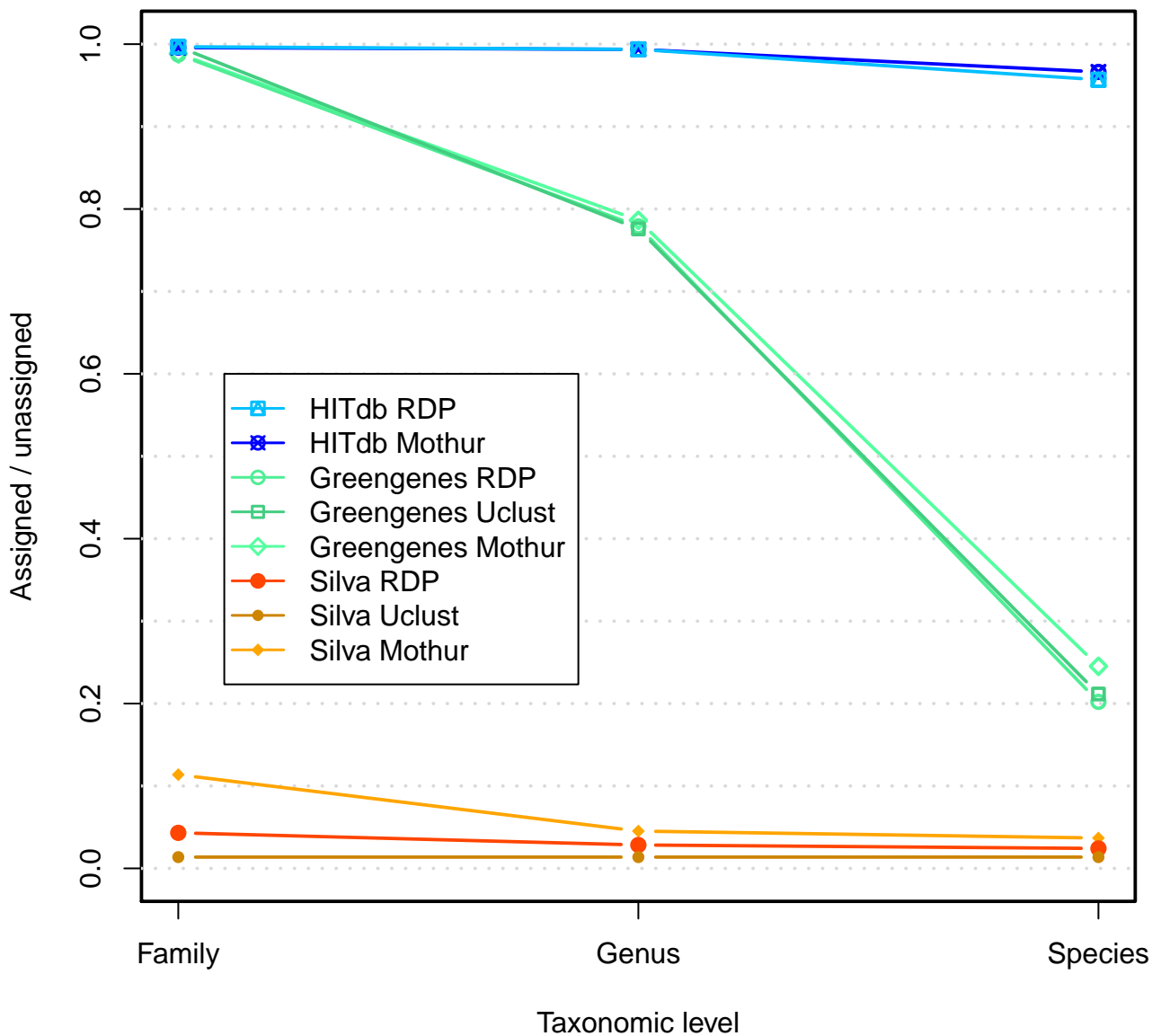

Supplement: Additional file 5: — Assignment of synthetic reads. The figure shows the relative numbers of assigned taxa for synthetic reads using Silva, Greengenes and HITdb databases, and RDP, Uclust and Mothur algorithms. (PDF 5 kb) [file 12864_2015_2265_MOESM5_ESM.pdf]

454

Proportion of sequences

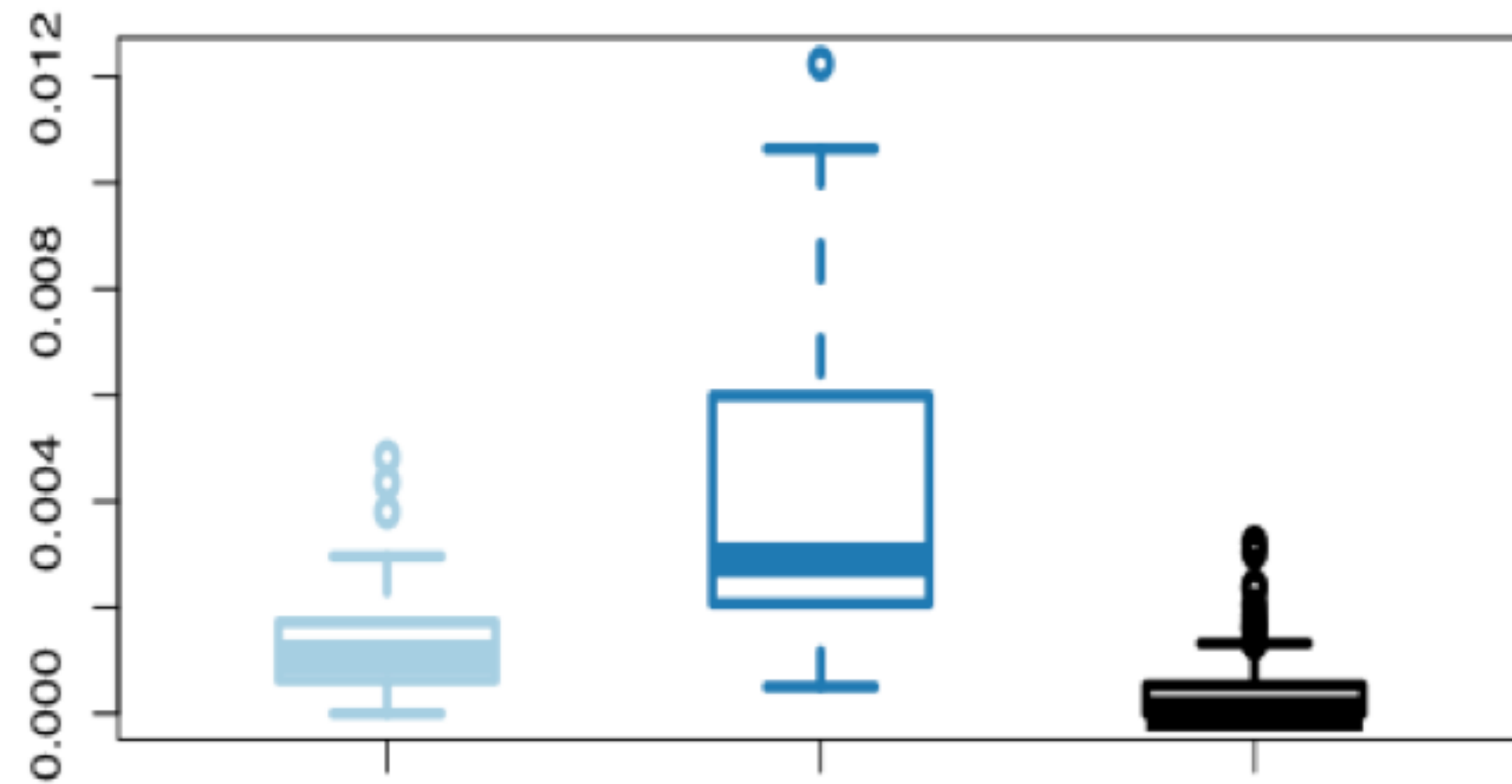

Illumina

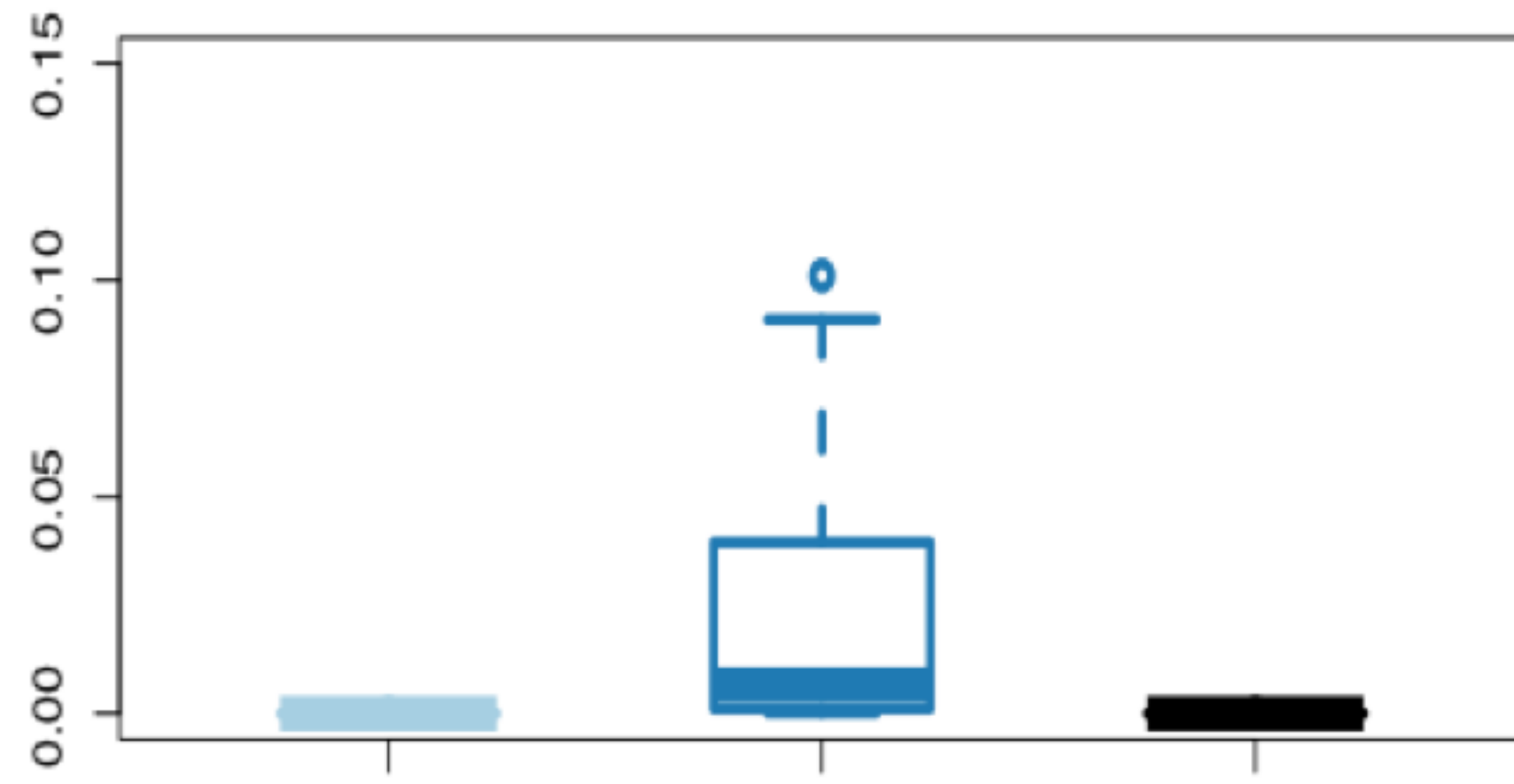

GG/RDP

GG/Uclust

HITdb/RDP

GG/RDP

GG/Uclust

HITdb/RDP

Supplement: Additional file 6: — Proportion of missing taxonomic assignments. The figure shows taxonomic assignments missing from Phylum level downwards in Greengenes and HITdb. The data is from two sets of biological samples sequenced either with 454 or Illumina MiSeq. (PDF 91 kb) [file 12864_2015_2265_MOESM6_ESM.pdf]

Processing time (s)

150  
50  
0

Memory usage (Gigabytes)

6  
5  
4  
3  
2  
1  
0

HITdb-RDP

GG+RDP

GG+Uclust

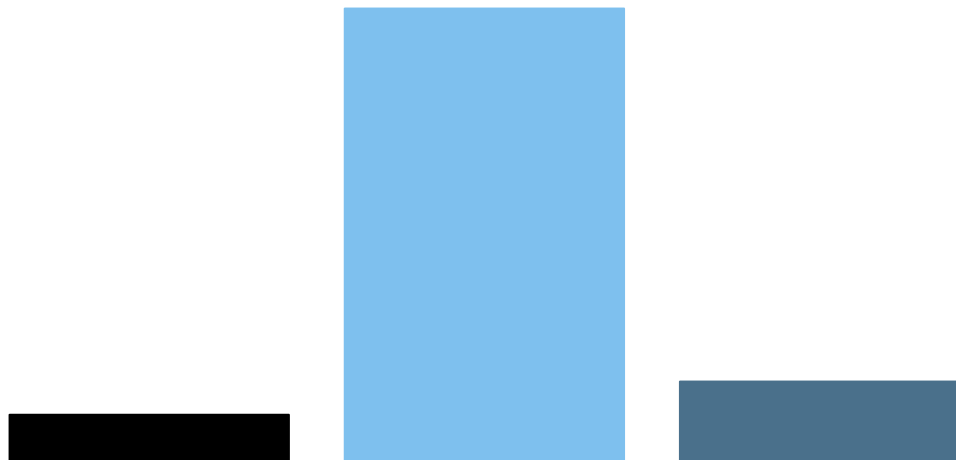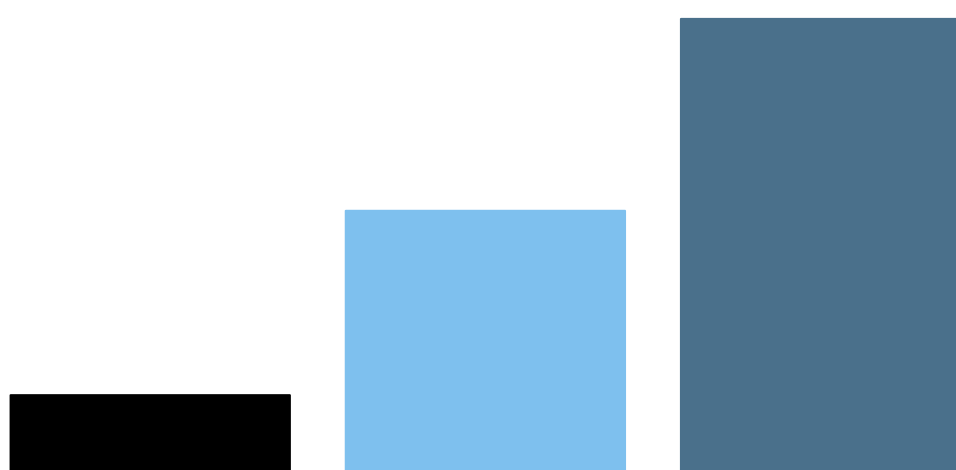

Supplement: Additional file 8: — Comparison of memory and time usage. The figure shows memory and time usage in HITdb and Greengenes databases. The measurement was done using 937 V4-V6 sequences from human intestinal bacterial species. (PDF 4 kb) [file 12864_2015_2265_MOESM8_ESM.pdf]
